# MRI-compatible soft fiber bioelectronics for multimodal assessment of electrical neural stimulation on whole-brain activation

**DOI:** 10.1093/nsr/nwag325

**Published:** 2026-05-28

**Authors:** Wenjun Li, Xiao Li, Haibo Yang, Chengqiang Tang, Zhenyu Wang, Qianfeng Wang, Yingnan Nie, Ziwei Liu, Yiqing Yang, He Wang, Songlin Zhang, Xiao-Yong Zhang, Shouyan Wang, Huisheng Peng, Xuemei Sun

**Affiliations:** State Key Laboratory of Molecular Engineering of Polymers, Department of Macromolecular Science, and Institute of Fiber Materials and Devices, Fudan University, Shanghai 200438, China; Institute of Science and Technology for Brain-Inspired Intelligence, Fudan University, Shanghai 200438, China; Neuromodulation and Brain-machine-interface Center, and MOE Frontiers Center for Brain Science, Fudan University, Shanghai 200438, China; Institute of Science and Technology for Brain-Inspired Intelligence, Fudan University, Shanghai 200438, China; Neuromodulation and Brain-machine-interface Center, and MOE Frontiers Center for Brain Science, Fudan University, Shanghai 200438, China; State Key Laboratory of Molecular Engineering of Polymers, Department of Macromolecular Science, and Institute of Fiber Materials and Devices, Fudan University, Shanghai 200438, China; Institute of Science and Technology for Brain-Inspired Intelligence, Fudan University, Shanghai 200438, China; Neuromodulation and Brain-machine-interface Center, and MOE Frontiers Center for Brain Science, Fudan University, Shanghai 200438, China; Institute of Science and Technology for Brain-Inspired Intelligence, Fudan University, Shanghai 200438, China; Institute of Science and Technology for Brain-Inspired Intelligence, Fudan University, Shanghai 200438, China; Neuromodulation and Brain-machine-interface Center, and MOE Frontiers Center for Brain Science, Fudan University, Shanghai 200438, China; State Key Laboratory of Molecular Engineering of Polymers, Department of Macromolecular Science, and Institute of Fiber Materials and Devices, Fudan University, Shanghai 200438, China; State Key Laboratory of Molecular Engineering of Polymers, Department of Macromolecular Science, and Institute of Fiber Materials and Devices, Fudan University, Shanghai 200438, China; Institute of Science and Technology for Brain-Inspired Intelligence, Fudan University, Shanghai 200438, China; State Key Laboratory of Molecular Engineering of Polymers, Department of Macromolecular Science, and Institute of Fiber Materials and Devices, Fudan University, Shanghai 200438, China; Institute of Pediatrics, Children’s Hospital of Fudan University, Shanghai 201102, China; Institute of Science and Technology for Brain-Inspired Intelligence, Fudan University, Shanghai 200438, China; Neuromodulation and Brain-machine-interface Center, and MOE Frontiers Center for Brain Science, Fudan University, Shanghai 200438, China; Institute of Science and Technology for Brain-Inspired Intelligence, Fudan University, Shanghai 200438, China; Neuromodulation and Brain-machine-interface Center, and MOE Frontiers Center for Brain Science, Fudan University, Shanghai 200438, China; State Key Laboratory of Molecular Engineering of Polymers, Department of Macromolecular Science, and Institute of Fiber Materials and Devices, Fudan University, Shanghai 200438, China; Institute of Pediatrics, Children’s Hospital of Fudan University, Shanghai 201102, China; State Key Laboratory of Molecular Engineering of Polymers, Department of Macromolecular Science, and Institute of Fiber Materials and Devices, Fudan University, Shanghai 200438, China; Institute of Pediatrics, Children’s Hospital of Fudan University, Shanghai 201102, China

**Keywords:** conductive polymer, fiber, flexible electronics, MRI‐compatible

## Abstract

Deciphering mechanisms of electrical neural stimulation using multimodal approaches combining electrophysiology and magnetic resonance imaging (MRI) is pivotal for advancing neuromodulation therapies. However, this paradigm has been hindered by the lack of high-performance neural electrodes that are compatible with ultra-high-field MRI while possessing exceptional electrochemical properties. Here, we report an MRI-compatible fiber neural electrode (MFE) fabricated from structurally optimized conductive polymer fiber emulating brain tissue characteristics. The MFE induces little-to-no MRI artifacts at 11.7 T and combines low modulus, low impedance and high charge‐injection limit, enabling precise neural stimulation and recording. Utilizing these MFEs, we investigated frequency-dependent whole-brain responses to electrical stimulation of the medial prefrontal cortex in wild-type and autism‐model rats, revealing responses potentially relevant to autism intervention. This was achieved through electrical stimulation synchronized with electrophysiological recording and multimodal MRI, including functional MRI, diffusion‐weighted imaging (tissue structural assessment) and magnetic resonance spectroscopy (metabolite profiling). Our MFE enables previously unattained simultaneous acquisition of multimodal information, providing a powerful tool for in-depth mechanistic studies of neuromodulation.

## Teaser

An MRI-compatible soft fiber neural electrode fabricated from conductive polymer is developed for multimodal exploration of neural electrical stimulation mechanisms through electrophysiological recording and MRI.

## INTRODUCTION

Neuromodulation technologies, particularly electrical stimulation with implantable electrodes, constitute the cornerstone for clinical treatment and study of neurological and psychiatric disorders [1–4]. Understanding the therapeutic mechanisms underlying electrical stimulation is critical for gaining valuable insights into complex brain functions [5,6]. As primary methods for these investigations, electrophysiological recording and magnetic resonance imaging (MRI) detect brain responses at distinct spatiotemporal resolutions, providing essential information from the local, neuronal‐cluster level to the whole‐brain level. MRI technology, in particular, has developed rapidly, evolving toward higher resolutions driven by increased magnetic field strengths (from 3 to 11.7 T) and enhanced versatility with advanced sequences including functional MRI (fMRI) [7], diffusion‐weighted imaging (DWI) [8] and magnetic resonance spectroscopy (MRS) [9]. These technologies enable comprehensive investigation of functional neural activity, tissue structure and metabolic processes in the brain [10–12].

However, the practical application of these advanced MRI technologies to study electrical neural stimulation remains limited, leading to a vague understanding of how stimulation influences large‐scale brain networks and ultimately hindering the clinical translation of these therapies [13]. A major obstacle is the absence of reliable and MRI‐compatible neural electrodes. Traditional metal-based neural electrodes (e.g., platinum–iridium alloy, PtIr) induce substantial magnetic field interference due to significant magnetic susceptibility mismatch with brain tissue. This generates imaging artifacts with sizes considerably larger than the electrodes themselves, obstructing accurate anatomical and functional mapping [14,15]. Furthermore, a pronounced mechanical mismatch exists between these rigid electrodes (modulus >100 GPa) and soft brain tissue (modulus <10 kPa), creating an unstable electrode–tissue interface that can provoke inflammation and compromise the electrode’s long-term functionality [16].

**Figure 1.**
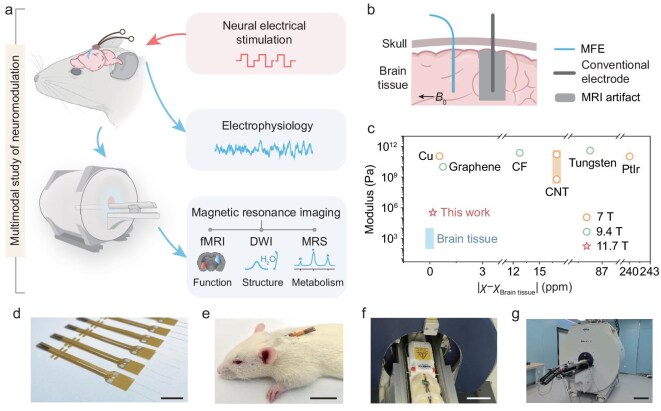
Multimodal investigation of neural modulation mechanisms with MFEs. (a) Schematic illustrating multimodal investigation of neuromodulation mechanisms through electrical stimulation synchronized with electrophysiological recording and multimodal MRI: fMRI, DWI and MRS. (b) Schematic of MRI artifacts induced by an MFE and a conventional MRI‐incompatible neural electrode. *B*_0_, static magnetic field. (c) Young’s modulus and magnetic susceptibility difference relative to brain tissue (|*χ*−*χ*_Brain tissue_|) for the electrode materials utilized in the MFE and previously reported neural probes for MRI studies. Data values are in Table S1. CNT: carbon nanotube; CF: carbon fiber. (d–g) Photographs of typical four-channel MRI-compatible neural devices with customized flexible printed circuit boards integrated with MFEs (d), a rat implanted with an MRI-compatible neural device (e) and the primary electrical stimulation-MRI setup (f, g). Scale bars: 1 cm (d), 5 cm (e, f), 30 cm (g).

While several efforts have been made to develop MRI-compatible electrodes [17–22] using materials like copper [17], various alloys [18] and carbon-based materials [19,20], these materials are still too stiff (with moduli of at least 600 MPa) to integrate seamlessly with soft brain tissue [23]. A few MRI-compatible film neural electrodes based on polymer substrates or hydrogels exhibit a low modulus, but most are limited to cortical applications [21,24]. Meanwhile, a small number of polymer fiber electrodes in deep brain still suffer from a relatively high modulus [25,26]. Most existing designs have demonstrated compatibility primarily in relatively low magnetic fields (1–9 T) and remained unvalidated in dedicated MRI investigations of neuromodulation mechanisms (Table S1). Therefore, there is an urgent need for soft neural electrodes compatible with high-field MRI to enable comprehensive studies of neuromodulation mechanisms through simultaneous electrical stimulation, electrophysiological recording and multimodal MRI (e.g., fMRI, DWI and MRS) for broader neuroscience applications (Fig. 1a).

Inspired by the brain’s extracellular matrix, which mainly consists of organic macromolecules (e.g., proteoglycan and fibrous collagen) and water [27], we have developed a soft, MRI-compatible fiber neural electrode (MFE) based on a conductive polymer gel fiber that exhibits negligible MRI artifact under ultra-high magnetic field up to 11.7 T (Fig. 1b). The MFE is constructed from poly(3,4‐ethylenedioxythiophene):poly(styrenesulfonate) (PEDOT:PSS) and has optimized magnetic susceptibility (−9.23 ppm) that closely matches that of brain tissue (−9.05 ppm), resulting in little-to-no artifact at 11.7 T (Fig. 1c). Meanwhile, the MFE exhibits the lowest modulus (328 kPa) among existing neural electrodes used for MRI studies (Fig. 1c and Table S1) and demonstrates outstanding electrochemical performance, including low impedance, high charge storage capacity (CSC) and a high charge injection limit (CIL) of 14.3 mC cm^−2^ (two orders of magnitude higher than that of PtIr). This enables both high-quality neural recording and spatially precise local neural stimulation *in vivo*. Utilizing these MFEs, we developed a flexible, MRI‐compatible neural interface device for multimodal interrogation of the electrical stimulation‐evoked effects in rat brain (Fig. 1d–g). We performed electrical stimulation synchronized with local field potential (LFP) recordings and multimodal MRI—including fMRI (related to functional neural activity), DWI (related to tissue structure) and MRS (related to metabolism)—at 11.7 T, which has not been achieved up to now. Through this approach, we revealed frequency‐dependent brain responses to medial prefrontal cortex (mPFC) stimulation in both autism spectrum disorder (ASD) model and wild-type (WT) rats, and observed regulatory effects of high-frequency electrical stimulation on ASD in certain brain regions. Overall, we present a universal tool and methodology for deconstructing neuromodulation mechanisms by means of electrophysiological recording and multimodal MRI. This electrode technology paves the way for designing advanced MRI-compatible bioelectronics and enabling future transformative studies in brain science.

## RESULTS AND DISCUSSION

### Fabrication and MRI compatibility of MFE

**Figure 2.**
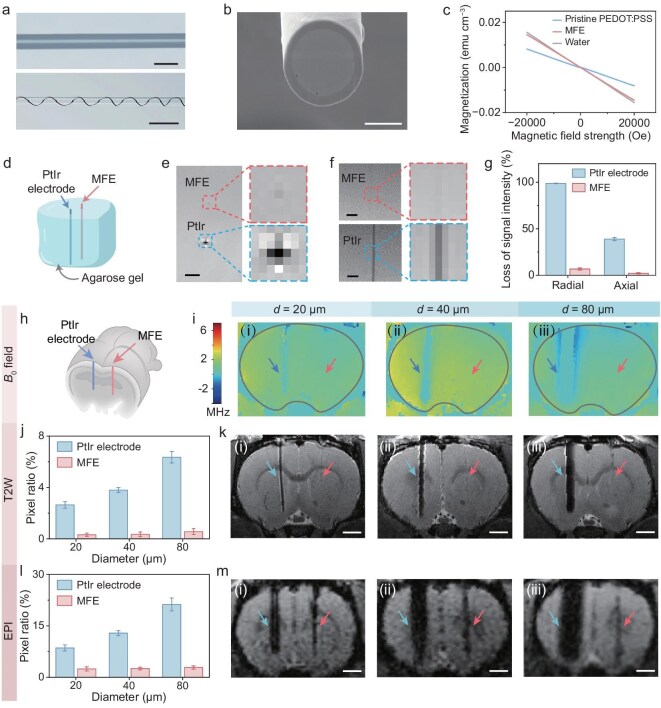
Morphology and MRI compatibility of MFE. (a) Top: optical microscopy image of a PEDOT:PSS fiber (∼15 μm in diameter). Bottom: photograph of a PEDOT:PSS fiber wrapped around a capillary. Scale bars: 20 μm (top), 2 mm (bottom). (b) Cross-sectional scanning electron microscopy image of the side-insulated MFE. Scale bar: 10 μm. (c) Magnetization curves of water, pristine PEDOT:PSS and the MFE. Water is commonly used as a reference to approximate the magnetic susceptibility of water-rich brain tissue. (d) Schematic of an MFE and a PtIr electrode implanted into agarose gel for MRI artifact assessment. (e, f) Representative radial (e) and axial (f) T2W images of agarose gel implanted with an MFE and a PtIr electrode. Scale bar: 1 mm. (g) T2W signal intensity loss around the electrodes (*n* = 3, mean ± standard deviation [SD]). (h) Schematic of an MFE and a PtIr electrode implanted contralaterally in the rat brain. (i) *B*_0_ inhomogeneity maps obtained from rat brains implanted with electrodes with diameters of 20, 40 and 80 μm. (j) Pixel ratios of T2W artifacts for MFEs and PtIr electrodes with different diameters (*n* = 4, mean ± SD). (k) Representative coronal T2W images of rat brains implanted with MFEs and PtIr electrodes with diameters of 20, 40 and 80 μm. Scale bar: 2 mm. (l) Pixel ratios of EPI artifacts for MFEs and PtIr electrodes with different diameters (*n* = 4, mean ± SD). (m) Representative EPI images of rat brains implanted with MFEs and PtIr electrodes with diameters of 20, 40 and 80 μm. Scale bar: 2 mm. Blue and red arrows in panels (i), (k) and (m) indicate PtIr electrodes and MFEs, respectively.

The MFEs were fabricated through wet spinning (Fig. S1a and b) of PEDOT:PSS suspension containing dimethyl sulfoxide (DMSO) with a coagulation bath composed of isopropyl alcohol (IPA) and DMSO. Glycerol was introduced into the fiber through an infiltration process to induce disorder and enhance flexibility [28]. The resulting fibers exhibited a uniform diameter of ∼15 μm (Fig. 2a). Compared to rapidly coagulated fibers from pure IPA, PEDOT:PSS fibers solidified gradually in DMSO/IPA co-solvent baths developed a porous internal architecture consisting of nanofibers (Fig. S1c). These fibers exhibit a Young’s modulus of ∼328 kPa, which is lower than that of fibers coagulated in pure IPA (Fig. S1d and e) and several orders of magnitude lower than those of previously reported metal- and carbon-based neural probes. This modulus more closely matches that of brain tissue (Table S1). As a highly polar aprotic solvent, DMSO incorporated in both PEDOT:PSS suspension and coagulation baths weakened the electrostatic interactions between PSS and PEDOT chains while solvating PSS segments. This dual action initiated the phase separation of PEDOT and PSS domains, facilitating the removal of excess insulating PSS. The PSS-to-PEDOT ratio characterized by X-ray photoelectron spectroscopy exhibited a progressive decrease across pristine PEDOT:PSS suspension, fibers prepared using pure IPA coagulation bath, and fibers prepared using DMSO/IPA mixed coagulation bath (Fig. S2a and b), resulting in an enhanced conductivity of 302 S cm^−1^ (Fig. S2c).

The diameter of PEDOT:PSS fiber could be tuned by twist‐assembling multiple primary fibers to accommodate various application requirements (Fig. S3a and b). During the infiltration process in the post-treatment solution (a mixture of water and glycerol in a 3:1 molar ratio), the polymer chain mobility was enhanced due to the solvent-induced swelling of the hydrophilic PSS phase and the plasticization effect of glycerol with high polarity and low volatility [29]. The twisted fibers fused into a monolithic architecture with a smooth surface free of demarcation lines and interfiber gaps through chain reorganization (Fig. S3c–e). The MFEs were coated with a polydimethylsiloxane (PDMS) layer for insulation (Fig. 2b), as PDMS was reported to be an MRI-compatible encapsulating material [15].

The spinning process also minimized the magnetic susceptibility difference between PEDOT:PSS and brain tissue/water (−9.05 ppm), resulting in an MFE diamagnetic susceptibility of −9.23 ppm (Fig. 2c). This enhanced diamagnetism arises from the conformation change in PEDOT from benzenoid coil structure to expanded quinoid structure (Fig. S4a) during the DMSO-induced phase separation. The more planar and delocalized quinoid segments are energetically more favorable for stabilizing bipolarons, resulting in a transition from spin-polarized polarons to spinless bipolarons in PEDOT, as confirmed by reduced electron paramagnetic resonance intensity (Fig. S4b and c) [30–33]. This conversion suppresses the spin (paramagnetic) contribution and makes the overall magnetic susceptibility more negative, i.e., an enhancement of diamagnetism. The magnetic susceptibility of the MFE, which is close to that of brain tissue, can lead to enhanced MRI compatibility.

We assessed MRI artifacts of the MFEs in an ultra-high-field 11.7 T MRI scanner and compared them with PtIr electrodes commonly used in clinical neural implants [34]. Prior to *in vivo* evaluation, we embedded MFEs and PtIr electrodes of identical diameter (∼15 μm) and insulating layer thickness (∼3 μm) in agarose gel that mimics brain tissue and imaged them (Fig. 2d). On T2-weighted (T2W) images, electrode-induced susceptibility artifacts manifested as hypointense regions. The MFEs produced no discernible artifacts at the current image resolution in both radial and axial T2W images (Fig. 2e and f), while PtIr electrodes generated prominent artifacts with a diameter of ∼345 μm. At the MFE implant sites, maximal signal losses relative to surrounding undisturbed regions were only 6.7% ± 1.2% (radial) and 2.2% ± 0.7% (axial). In contrast, PtIr electrodes exhibited signal losses of 98.8% ± 0.3% (radial) and 38.9% ± 1.7% (axial) (Fig. 2g and Fig. S5). These results demonstrate that the MFEs induce substantially smaller artifacts and reduce signal loss, preserving MRI image integrity and signal-to-noise ratio.

Prior to *in vivo* MRI artifact assessment, we evaluated the biocompatibility of MFEs by analyzing immune cell distribution around the electrode following chronic implantation. The MFE was implanted into brain tissue with minimal invasiveness via a tungsten wire-assisted approach, inducing no significant tissue damage (<100 μm) at the implantation site (Fig. S6). This minimal injury profile provides a foundation for reducing immune responses. Fluorescence imaging and quantitative analysis revealed minimal astrocyte/microglia activation and negligible neuronal loss in MFE-implanted brains, at levels comparable to those of non-implanted controls and lower than those observed with rigid PtIr electrodes. These findings confirm the excellent biocompatibility of the MFEs (Fig. S7).

The MFEs and PtIr electrodes were then implanted contralaterally in rat brains to assess MRI artifacts *in vivo* (Fig. 2h). Given that neuromodulation of nuclei with varied geometries requires electrodes with different dimensions, we tested electrodes with diameters of ∼20, 40 and 80 μm. Electrodes with identical exposed cross-sectional areas and insulation layers were implanted in brain tissue with the same orientation (Figs S8 and S9). Static magnetic field (*B*_0_) maps (Fig. 2i) showed no detectable distortions surrounding the MFEs of any diameter, whereas PtIr electrodes introduced substantial *B*_0_ interference that scaled with electrode size. A similar trend was observed in radiofrequency magnetic field (*B*_1_) maps (Fig. S10a). In both coronal (Fig. 2j and k) and horizontal (Fig. S10b) T2W images, MFE artifacts were scarcely visible with dimensions comparable to the imaging resolution and did not exhibit significant enlargement as the electrode diameter increased, in contrast to the artifacts observed for PtIr electrodes. Using an automated artifact area detection method to quantify pixels affected by electrode artifacts (Fig. S11), we determined that MFE pixel ratios (the percentage of the pixels occupied by artifacts) were 0.32% ± 0.13%, 0.35% ± 0.18% and 0.56% ± 0.23% for 20, 40 and 80 μm electrodes, respectively, ∼10 times lower than those of PtIr electrodes (2.6% ± 0.3%, 3.8% ± 0.2% and 6.4% ± 0.4%) (Fig. 2j).

Echo-planar imaging (EPI), a sequence widely used in fMRI, is more sensitive to magnetic field distortions from implants, resulting in larger artifacts than those observed in T2W images (Fig. 2l and m). As electrode diameter increased from 20 to 80 μm, pixel ratios of MFEs artifacts increased modestly from 2.4% ± 0.6% to 2.9% ± 0.5%, whereas PtIr electrodes exhibited progressively more severe artifacts, with pixel ratios rising from 8.6% ± 0.9% to 21.3% ± 1.9% (Fig. 2l). The maximum artifact diameter for an 80 μm MFE was ∼0.3 mm in EPI images and ∼0.1 mm in T2W images, substantially smaller than those of PtIr electrodes (∼2.7 mm in EPI images and ∼1.0 mm in T2W images) (Fig. S12). Sequential rostral-to-caudal MRI slices showed that PtIr artifacts spanned three slices in T2W images and at least four slices in EPI images, whereas MFE artifacts were visible only in a single slice (Figs S13 and S14).

To enable more precise comparison, we expanded the artifact assessment to include MFE and PtIr electrodes with larger diameters of 100 and 200 μm. The MFE artifacts became more readily discernible in this assessment. We quantitatively analyzed the artifact-to-electrode diameter ratio across all electrode diameters (Fig. S15). The artifact-to-electrode diameter ratios of all PtIr electrodes were higher than those of MFE, and this disparity widened progressively with increasing diameter. At the point of maximum divergence, the ratio for PtIr electrodes reached nearly 10 times that of MFE. Notably, MFE artifacts remained negligible following long-term implantation (Fig. S16). This substantial reduction in artifacts enables unobstructed anatomical and functional brain visualization in MRI for extended neuroscience studies.

### Electrochemical performance and neural interfacing capabilities of **MFE**

**Figure 3.**
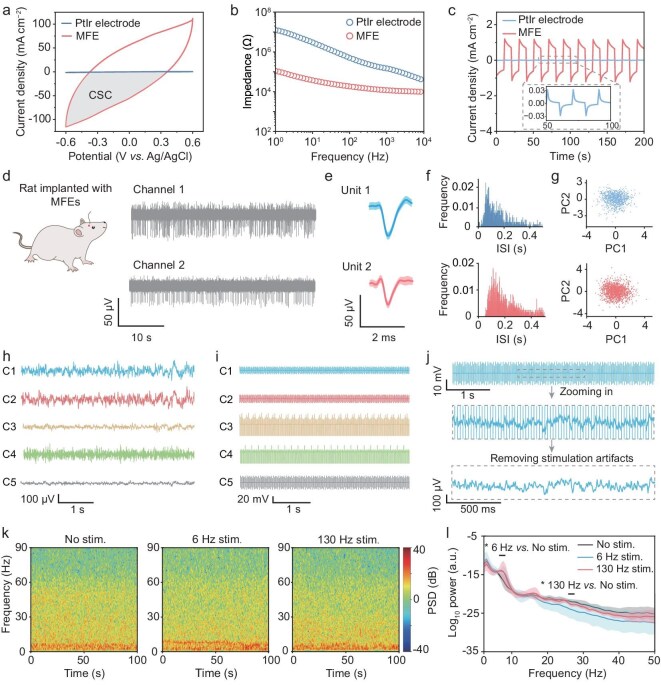
Electrochemical performance and neural interfacing capabilities of MFE. (a) Typical cyclic voltammograms of MFE and PtIr electrode. The time integral of the negative current shown by the shadow region represents the CSC. (b) Impedance of MFE and PtIr electrode (20 μm in diameter). (c) Current outputs under ±0.5 V bipolar pulses (50 Hz). (d) Representative band-pass-filtered neural waveforms recorded via dual-channel MFEs in the rat hippocampus. (e) Sorted single-unit spikes from channel 1 (top) and channel 2 (bottom). (f) ISI distributions for channels 1 (top) and 2 (bottom). (g) PCA of spikes from channels 1 (top) and 2 (bottom). (h) LFPs recorded without stimulation. C1–C5 denote five recording channels. (i) LFPs recorded during 130 Hz stimulation. (j) Schematic of stimulation artifact removal. (k) Time-frequency representations of LFPs under no stimulation, 6 Hz stimulation and 130 Hz stimulation. (l) LFP power spectra (*n* = 5; two-tailed paired sample *t*-test, *P < 0.05). Curves show means; shading indicates SD.

Electrochemical performance is a critical indicator for evaluating neural electrodes. Due to the inherent mixed ionic-electronic conduction capability of PEDOT:PSS and the porous architecture within the fiber electrode, there are abundant internal interfaces that facilitate efficient charge storage and transfer. Therefore, the MFE exhibits a substantially higher normalized electroactive surface area than commonly used PtIr electrodes (Fig. S17a and b), reflecting a larger accessible surface area, which is beneficial for high volumetric capacitance and low interfacial impedance [28]. CSC describes how much charge an electrode can store and deliver across the electrode–electrolyte interface under a given potential [35,36]. The CSC of the MFE (1420 mC cm^−2^) is approximately two orders of magnitude higher than that of PtIr electrode (12.5 mC cm^−2^) (Fig. 3a and Fig. S17c). Owing to its high surface area and capacitance [37], the MFE (20 μm in diameter) exhibits an impedance of 11.9 kΩ at 1 kHz (Fig. 3b), an order of magnitude lower than that of the PtIr electrode with the same diameter (148 kΩ at 1 kHz). Lower impedance at the electrode–electrolyte interface facilitates more efficient charge transfer, resulting in higher signal fidelity and lower noise during neural recording and stimulation [38,39].

Leveraging both Faradaic and non-Faradaic mechanisms at the electrode–electrolyte interface [20,40], the MFE exhibits a markedly higher charge injection capability. At the same applied voltage, the MFE produced a 40-fold higher current than the PtIr electrode (Fig. 3c). To quantify this, we measured CIL, which is defined as the maximum charge injected during a current-controlled stimulation pulse without polarizing the electrode beyond the potential for water reduction or oxidation [40]. We used a negative polarization potential of −0.6 V as the negative polarization threshold (Fig. S17d and e) [41]. The MFE exhibited a CIL of 14.3 mC cm^−2^, significantly exceeding the 0.121 mC cm^−2^ of PtIr electrode and surpassing other electrode materials such as titanium nitride, carbon nanotube fiber and graphene fiber (Fig. S17f). High CIL enables equivalent stimulation currents at lower voltages with smaller electrodes, concentrating stimulation within a highly localized region and thereby minimizing off-target effects caused by broad-area stimulation [42]. High CIL also expands the safe stimulation window and enhances stimulation safety.

Furthermore, the electrochemical properties of the MFE remained stable under both mechanical and physiological stresses. After 100 bending cycles, 10^5^ current pulses and 2-week immersion in phosphate-buffered saline, the impedance, CSC and CIL values exhibited negligible changes (Fig. S17g–i), confirming the robustness of the MFE under repeated mechanical deformation, prolonged electrical stimulation and physiological conditions. In addition to the intrinsic stability of the electrode, potential protein fouling during implantation could affect the impedance stability at the electrode–tissue interface. Due to the infiltration of the post-treatment solution within the fiber matrix and the relatively high content of the hydrophilic component PSS, the MFE exhibits a hydrophilic surface, which is beneficial for mitigating non-specific protein fouling. No significant increase in impedance was observed for the MFE before and after immersion in a 10 mg mL^−1^ bovine serum albumin solution for 1 h (Fig. S18a). Over 30 days of brain implantation, the impedance of the MFE showed only a slight increase (Fig. S18b and c), indicating its ability to form a stable interface with brain tissue. Considering the requirements for MRI applications, we evaluated the impedance and charge injection behavior of the MFE during multiple MRI sequences. The results were consistent with those obtained under non-magnetic conditions, further validating the electrochemical stability of MFE (Fig. S19). Under identical voltage stimulation, the slight increase in the observed current is likely attributable to magnetohydrodynamic-enhanced ion transport under the strong static magnetic field, as well as weak additional currents induced by the time-varying magnetic fields [43,44].

Given the low impedance, high CSC and large CIL, the MFE is a promising candidate for high-quality neural recording and stimulation. We conducted neural recordings in the rat hippocampus using MFEs (Fig. 3d). Two single-unit signals were successfully detected and sorted from two MFE channels, with high signal-to-noise ratios, well-defined interspike interval (ISI) distributions and clear separation in principal component analysis (PCA) space (Fig. 3e–g). These results demonstrate that the MFE enables high-fidelity neural recordings. Furthermore, we demonstrated effective neural electrical stimulation with simultaneous neural feedback recording using MFEs in the mPFC of WT rats. We removed the stimulation artifacts from LFPs using an irregular-sampling method (Fig. 3h–j) and observed frequency-dependent stimulation effects. Low-frequency (6 Hz) and high-frequency (130 Hz) electrical stimulation produced immediate and distinct modulation effects on neural oscillations (Fig. 3k). In the LFP power spectrum, the power in the 6–8 Hz band increased significantly during 6 Hz stimulation, whereas the power in the 25–28 Hz band was significantly suppressed during 130 Hz stimulation (Fig. 3l). These distinct modulation effects demonstrate the capability of the MFE to investigate mechanisms of electrical neural stimulation in an electrophysiological manner and highlight its potential applications in diverse neurophysiological research.

### Simultaneous electrical neural stimulation and multimodal MRI in ASD model rats

Leveraging the MRI compatibility and neural recording/stimulation capabilities of the MFE, we conducted simultaneous electrical neural stimulation and multimodal MRI at 11.7 T in a rat model of ASD. ASD is a neurodevelopmental disorder with a high incidence rate [45,46], and there is currently no fully effective clinical treatment. Previous studies in *MECP2*-duplication rat models of ASD revealed abnormal LFP characteristics in mPFC [47,48], and mPFC-specific *MECP2* knockdown rescued social behavior deficits [49], suggesting that the mPFC is a feasible intervention target for ASD. By implanting MFEs in the mPFC, we explored how electrical stimulation at different frequencies (130 Hz for high frequency, 6 Hz for low frequency) affects global brain activity in *MECP2* transgenic (TG) rat models using multimodal MRI, including fMRI, DWI and MRS. To facilitate multimodal MRI, we fabricated MRI‐compatible neural devices by integrating MFEs with a custom flexible printed circuit that relocates the connection between electrodes and the external circuitry from the head to the dorsal side (Fig. S20), allowing a four-channel receive coil to be placed close to the scalp, which is essential for achieving high-quality MRS.

**Figure 4.**
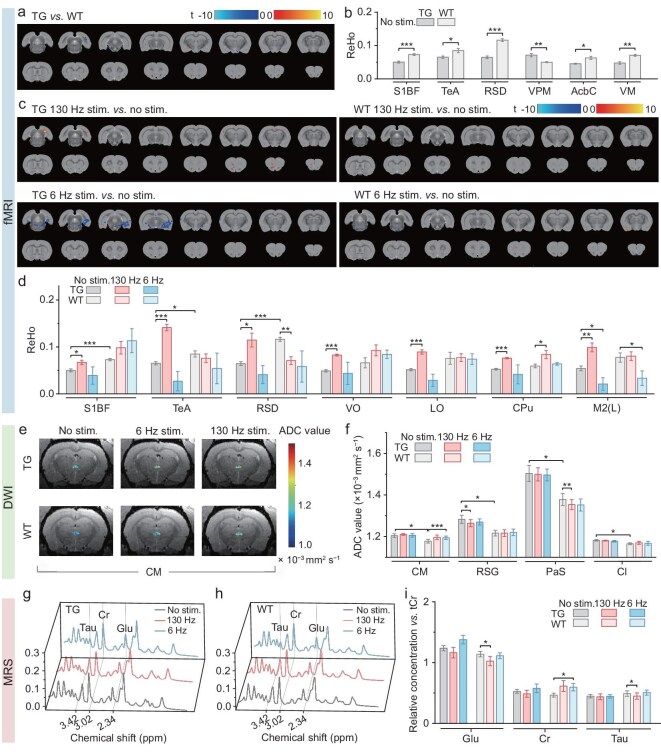
Simultaneous electrical neural stimulation and multimodal MRI with MFEs at 11.7 T in a rat model of ASD. (a) ReHo change maps showing the differences in ReHo values between TG and WT rats at a significance threshold of *P* < 0.01 (TG vs. WT; two-tailed *t*-test; *n* = 5 for TG, *n* = 6 for WT; red: TG > WT, blue: TG < WT). (b) ReHo quantification in specific brain regions of TG and WT rats (mean ± standard error of the mean [SEM]; *n* = 5 for TG, *n* = 6 for WT; two-tailed *t*-test). AcbC: accumbens nucleus, core; VM: ventromedial thalamic nucleus. (c) ReHo change maps showing the ReHo value differences between TG and WT rats under no stimulation and electrical stimulation at 130 Hz or 6 Hz. The significance threshold is *P* < 0.01 (130 Hz or 6 Hz stimulation vs. no stimulation; two-tailed *t*-test; *n* = 5 for TG, *n* = 6 for WT). (d) ReHo quantification in specific brain regions of TG and WT rats during no stimulation, 130 Hz stimulation and 6 Hz stimulation (mean ± SEM; *n* = 5 for TG, *n* = 6 for WT; two-tailed *t*-test). M2(L): secondary motor cortex (left). (e) Representative ADC maps of CM. (f) ADC values in specific brain regions of TG and WT rats during no stimulation, 130 Hz stimulation and 6 Hz stimulation (mean ± SEM; *n* = 7 for TG, *n* = 8 for WT; two-tailed paired sample *t*-test). (g, h) Linear combination model fits of MRS spectra acquired from the mPFC in TG (g) and WT rats (h). Peaks at 2.34, 3.02 and 3.42 ppm correspond to Glu, Cr and Tau, respectively. (i) Metabolite concentrations normalized to total creatine (tCr) (mean ± SEM; *n* = 7 for TG, *n* = 8 for WT; two-tailed paired sample *t*-test). *P < 0.05, **P < 0.01, ***P < 0.001, unlabeled bars indicate no significant difference (ns, *P* > 0.05).

Through regional homogeneity (ReHo, quantifying the local synchronization of neural activity) analysis of fMRI data, we assessed the blood oxygen level-dependent responses in TG rats and WT controls during mPFC stimulations at different frequencies. The results revealed that *MECP2* duplication led to abnormal activity across multiple brain regions (Fig. 4a and b), consistent with prior fMRI studies [49].

In TG rats, 130 Hz mPFC stimulation significantly increased ReHo in brain regions previously established to be associated with *MECP2* duplication-induced aberrant brain function and structural alterations, including the barrel field of primary somatosensory cortex (S1BF), temporal association cortex (TeA), retrosplenial dysgranular cortex (RSD), ventral orbital cortex (VO), lateral orbital cortex (LO), caudate putamen (CPu) and secondary motor cortex [50–53]. Notably, 130 Hz stimulation induced increases in ReHo that counteracted the ReHo decreases induced by *MECP2* duplication in S1BF, TeA and RSD of TG rats (Fig. 4c and d). This counteractive effect was not observed in WT controls. Given the established association between these regions and ASD pathogenesis [54–57], our findings suggest that 130 Hz mPFC stimulation has the potential to modulate ASD-relevant neural activity and identify these regions as candidate targets for ASD intervention.

Frequency-dependent brain responses were also induced by mPFC stimulation in WT rats (Fig. S21). Notably, 130 Hz stimulation significantly increased ReHo in RSD but decreased it in CPu, while 6 Hz stimulation increased ReHo in regions including hippocampus CA2 and the anterior part of basolateral amygdaloid nucleus. Both 130 and 6 Hz stimulation influenced neural activity in the prelimbic cortex (PrL), ventral posteromedial thalamic nucleus (VPM) and area 1 of cingulate cortex in WT rats. These results demonstrate that mPFC stimulation elicits frequency-dependent activity patterns in both TG and WT rats, reflecting complex functional connectivity in the brain.

In addition to fMRI, we performed stimulation‐synchronized MRI with additional modalities to validate the universality of MFEs. DWI is an MRI sequence that exploits water diffusion properties to probe tissue characteristics, white‐matter structure and microvascular perfusion [58–60]. Elevated apparent diffusion coefficient (ADC) values typically reflect enhanced water diffusion in tissues. Simultaneous mPFC stimulation and DWI revealed significant differences in ADC values between TG and WT rats in multiple brain regions, including the central medial thalamic nucleus (CM), retrosplenial granular cortex (RSG), parasubiculum (PaS) and claustrum (Cl) (Fig. 4e and f and Fig. S22a–c). Notably, in the RSG, 130 Hz mPFC stimulation in TG rats reversed *MECP2* duplication-induced changes in ADC (Fig. 4f). This stimulation also increased ADC values in the ventral tegmental area and primary somatosensory cortex hindlimb region (S1HL) in TG rats (Fig. S22d, e and h) but decreased ADC values in the PaS and S1HL of WT rats (Fig. 4f and Fig. S22h). Low-frequency (6 Hz) stimulation significantly increased ADC in the rhomboid thalamic nucleus in both TG and WT rats, and in the CM and ventral hippocampal commissure in WT rats (Fig. 4f and Fig. S22f–h). Unlike fMRI, DWI provides complementary insights into mPFC stimulation effects by capturing tissue microstructure and diffusion dynamics.

Finally, we performed MRS to non-invasively measure metabolite changes at the stimulation site (mPFC) (Fig. 4g and h). WT rats exhibited frequency-dependent changes during stimulation, while electrical stimulation induced no significant metabolite alterations in TG rats. Stimulation of 130 Hz significantly reduced glutamate (Glu) concentration (Fig. 4i), indicating suppressed excitatory neurotransmission. Concurrently, taurine (Tau) concentration was significantly reduced. As an inhibitory neurotransmitter, Tau reduces oxidative stress, inflammation and mitochondrial dysfunction, while protecting against Glu excitotoxicity in the central nervous system [61], demonstrating its neuroprotective effects. The decrease in Tau resembles MRS findings of 130 Hz deep brain stimulation-induced Tau reduction in Parkinson’s disease [62] and parallels decreased Tau levels observed after pharmacological depression treatment [63]. Therefore, high-frequency neural electrical stimulation increases Tau turnover, potentially contributing to neuroprotection and neuroplasticity [64,65]. Conversely, 6 Hz electrical stimulation significantly increased creatine (Cr) concentration (Fig. 4i), indicating attenuated energy metabolism.

Through concurrent mPFC stimulation using MFEs and multimodal MRI including fMRI, DWI and MRS, we comprehensively characterized the whole-brain effects of electrical stimulation from multiple perspectives related to neural function, tissue microstructure and metabolic changes. High-fidelity, artifact-free MRI images ensured precise assessment of brain responses in perielectrode regions (e.g., the PrL). Frequency-dependent responses revealed that high-frequency (130 Hz) mPFC stimulation holds therapeutic potential by modulating neural activity in ASD-relevant regions, providing new insights into ASD modulation mechanisms. Furthermore, throughout the 15-min MRI procedure using the sequence applied in this study, temperature increases at the electrode tips in the brain tissue-mimicking gel remained below 1°C (Fig. S23), confirming that the MFEs were free from radiofrequency-induced heating risks. This further validates the MRI compatibility and application potential of the MFE.

Although simultaneous neural electrical stimulation and fMRI were demonstrated for mechanistic studies, achieving simultaneous electrophysiological recordings with fMRI is equally critical for comprehensive mechanistic understanding. This integration has not yet been implemented in this study. Future efforts should focus on improved shielding and filtering strategies, precise synchronization of electrophysiological recording with MRI pulse sequences, and advanced signal artifact removal algorithms to enable reliable simultaneous integration of electrophysiology recording and fMRI.

## CONCLUSION

In summary, we developed a flexible, MRI-compatible fiber neural device based on conductive polymer fiber and demonstrated its utility as a practical tool for multimodal exploration of electrical stimulation mechanisms. Superior to previously reported MRI-compatible electrodes, the MFE combines low modulus, exceptional electrochemical performance (low impedance, high CSC and high CIL), and critically, minimal MRI artifacts under ultra-high magnetic field strength (11.7 T), due to its magnetic susceptibility being close to that of brain tissue. Furthermore, we applied this device in multimodal investigations to study neuromodulation effects of electrical stimulation through electrophysiological recording and MRI (fMRI, DWI and MRS), revealing the potential of high-frequency mPFC electrical stimulation in modulating ASD-related neural activity. This approach enables comprehensive mapping of stimulation‐induced brain response patterns, offering new insights into mechanisms of electrical neuromodulation. Ultimately, the MFE presents a versatile methodology for investigating the therapeutic mechanisms underlying various neural stimulation therapies. This electrode technology can be broadly applied to fabricate diverse MRI-compatible bioelectronics, paving the way for advances in neuroscience research.

## Supplementary Material

nwag325_Supplemental_File

## METHODS

Details of the materials and methods are available in the online supplementary file.
