# Supplementary material for: MRI-compatible soft fiber bioelectronics for multimodal assessment of electrical neural stimulation on whole-brain activation
Source: Natl Sci Rev. 2026 May 28;13(13):nwag325. doi: 10.1093/nsr/nwag325 (PMC13358296; doi:10.1093/nsr/nwag325)
Supplement: nwag325_Supplemental_File [file nwag325_supplemental_file.pdf]

Supplementary Information for

MRI-compatible soft fiber bioelectronics for multimodal assessment of electrical neural stimulation on whole-brain activation

Wenjun Li[1,†], Xiao Li[2,3,†], Haibo Yang[2,3,†], Chengqiang Tang[1], Zhenyu Wang[2,3], Qianfeng Wang[2], Yingnan Nie[2,3], Ziwei Liu[1], Yiqing Yang[1], He Wang[2], Songlin Zhang[1,4], Xiao-Yong Zhang[2,3,*], Shouyan Wang[2,3,*], Huisheng Peng[1,4,*] and Xuemei Sun[1,4,*]

[1]State Key Laboratory of Molecular Engineering of Polymers, Department of Macromolecular Science, and Institute of Fiber Materials and Devices, Fudan University, Shanghai 200438, China;

[2]Institute of Science and Technology for Brain-Inspired Intelligence, Fudan University, Shanghai 200438, China;

[3]Neuromodulation and Brain-machine-interface Center, and MOE Frontiers Center for Brain Science, Fudan University, Shanghai 200438, China;

[4]Institute of Pediatrics, Children's Hospital of Fudan University, Shanghai 201102, China

***Corresponding authors. E-mails:** sunxm@fudan.edu.cn; penghs@fudan.edu.cn; shouyan@fudan.edu.cn; zhangxiaoyong@sjtu.edu.cn
[†]Equally contributed to this work.

This file includes:

Methods (Page S2–S7)
Figure S1 to S23 (Page S8–S31)
Table S1 (Page S32–S34)

Methods

Preparation of MRI-compatible fiber neural electrodes (MFEs)

PEDOT:PSS suspension (PH1000, Heraeus) was concentrated at 90ºC until reaching 2.5% solid content, followed by adding 5 wt% dimethyl sulfoxide (DMSO, Sinopharm Chemical Reagent Co., Ltd). The mixture was loaded into a 1 mL syringe mounted on an injection pump (LSP02-2A, Longer Precision Pump Co., Ltd) and extruded into a coagulation bath (isopropyl alcohol/DMSO = 9:1 *v*/*v*) through a 32-gauge spinneret at a flow rate of 10 μL min^{-1}. After washing with water, drying, and infiltrating with a water/glycerol mixture (3:1 molar ratio) for 1 h, the resulting MFEs were obtained. For insulation, a polydimethylsiloxane precursor (SYLGARD184, Dow Corning) mixture (A:B = 1:9) diluted with n-octane [TCI (Shanghai)] was prepared (PDMS: n-octane 3:7 *w*/*w*). Then the MFE was coated by scraping the fiber with a 1 μL bead of PDMS solution at a constant speed and cured at 70°C for 20 min. The MFEs were cut to the desired length, and their cross sections were exposed as active sites. Pristine PEDOT:PSS membranes were prepared by spin-coating and drying (40°C) of PEDOT:PSS suspension.

Characterization of MFEs

Scanning electron microscope (SEM) images were obtained by Zeiss Gemini SEM500. Measurements of the Young's modulus were conducted by HY0350 table-top universal testing instrument. Magnetic susceptibility tests were conducted by Vibrating sample magnetometer (LakeShore, USA) at 25°C. The X-ray photoelectron spectroscopy (XPS) was recorded on Thermo Scientific K-Alpha.

Electrical resistance (R), diameter (d), and length (L) were measured by the Keithley 2410, Olympus EX51, and vernier caliper, respectively. PEDOT:PSS fibers were placed straight on the glass sheet, and the silver glue was used to connect fibers to detection probes. Electrical conductivity (σ) was calculated according to R, d and L.

$$\sigma = \frac{1}{R} * \frac{4L}{\pi * d^2}$$

All electrochemical measurements were performed in 1× phosphate-buffered saline (PBS, pH 7.4). Electrochemical impedance spectroscopy (EIS) and cyclic voltammograms (CV) were conducted on a CHI 660E electrochemical workstation using a three-electrode configuration, where the tested sample, an Ag/AgCl electrode, and a platinum wire served as the working, reference, and counter electrodes, respectively. EIS was measured via AC impedance over a frequency range of 1–10^4 Hz. CV was performed over a voltage range of −0.6 to 0.6 V at a scan

rate of 0.05 V s^{-1}. The CSC was calculated by integrating the cathodic current over the same potential range and normalizing to the geometric surface area of the exposed fiber cross-section. For voltage transient measurements, a three-electrode configuration was employed. Biphasic, symmetric, charge-balanced current pulses (300-μs pulse width, 60-μs inter-phase delay) were delivered to the tested sample via a stimulator (Model 3800, A-M Systems, USA). Voltage transients were recorded with CHI 660E using open-circuit potential program. The CIL was evaluated by acquiring voltage transients at increasing current amplitudes until the electrode polarization potential (E_p) reached the water electrolysis threshold (−0.6 V). The cathodic CIL was calculated as the total cathodic-phase charge divided by the geometric surface area of the exposed fiber cross-section. The electrodes were kept in PBS and tested weekly to assess long-term stability. Stability under continuous current pulsing was evaluated in a two-electrode configuration with the MFE as the working electrode and a platinum wire as the counter electrode. Testing after 10^5 stimulation cycles (130 Hz, biphasic, symmetric charge-balanced current pulses, 60-μs phase duration) at 500 μA amplitude.

Animals

The animal experiments were approved by the Institutional Animal Care and Use Committee at Fudan University (Approval No. 2022120057Z). Rats (Sprague Dawley, 8 weeks old, male) for electrophysiological recording were purchased from Shanghai SLAC Laboratory Animal Co. Ltd. (Certificate Number: SYXK-Hu-2022-0012). All procedures followed guidelines described by the National Institutes of Health and Fudan University. Autism model rats were constructed via artificial chromosome recombination [1]. WT rats in the same litter were used as controls. All implantation surgeries were performed when the weight of rats reached 350–400 g, with anesthesia induced by 2.5% concentration of isoflurane. After a 7-day recovery period, the rats underwent subsequent experiments.

Immunofluorescence staining

Animals were euthanized and the brain tissue was carefully removed. The brain was fixed in 4% paraformaldehyde for 24 h and subjected to gradient ethanol dehydration. The dehydrated tissues were embedded in paraffin and sliced with a microtome to a thickness of 4 μm. Finally, the slices were dried at 60 °C for 2 h. The sections underwent H&E and immunofluorescence staining following standard procedures. For neuron and astrocyte labeling, anti-neuronal nuclei protein antibodies (anti-NeuN, 1:500, GB13138-1, Servicebio) and anti-glial fibrillary acidic protein antibodies (anti-GFAP, 1:1000, GB11096, Servicebio) were employed as the primary antibodies. Secondary antibodies included Cy3 conjugated Goat Anti-Rabbit lgG (H+L) (1:300, GB21303, Servicebio) and Alexa Fluor® 488-conjugated Goat Anti-Mouse lgG (H+L) (1:500,

GB25301, Servicebio). For neuron and microglia labeling, the primary antibody was replaced with anti-ionized calcium binding adaptor molecule-1 antibodies (anti-Iba-1, 1:200, GB13105-1, Servicebio), and other antibodies were the same. Immunofluorescence sections were mounted with DAPI (G1012, Servicebio).

***In vivo* electrophysiology**

For electrophysiological recordings, MFEs with a diameter of ~20 μm (15-μm PEDOT:PSS fiber and 3-μm-thick insulator) were connected to a commercial connector using silver glue, with joints sealed by silicone rubber. Copper wires served as reference (Ref) and ground (GND) electrodes. MFEs were adhered parallel and adjacently to a 100-μm diameter tungsten wire (implantation aid) with their recording terminals aligned using gelatin solution, followed by air-drying at room temperature and UV sterilization for later use. The distance between their recording point centers was comparable to their diameter, approximately 20 μm. The gelatin solution was prepared by dissolving 5 g gelatin in 10 g saline at 120°C. The MFE-bearing tungsten wire was implanted into the rat hippocampus (AP: −3.5 mm, ML: +2.5 mm, DV: −3.5 mm) through stereotactic surgery. Following 15-minute dissolution of gelatin, the tungsten wire was slowly retracted from the brain tissue using the stereotaxic apparatus, leaving the MFEs deposited at the target location. The Ref and GND wires were connected to occipital bone anchor screws. Skull drill holes were sealed with tissue glue, and the entire electrode was embedded and fixed by dental cement with the insertion port exposed. Recordings were performed using a CerePlex Direct system (Blackrock Microsystems). Signals were band-pass filtered (300–3000 Hz), with spikes sorted and analyzed in Spike2 software.

For stimulation-synchronous local field potential (LFP) recordings, two symmetrical MFE bundles—each comprising three MFEs (~40-μm diameter) with tips arranged in a 500-μm gradient spacing—were connected to commercial Samtec connector. Two copper wires served as Ref/GND and stimulation return electrodes. The middle MFE in one bundle delivered stimulation while others recorded signals. MFEs were implanted in rat mPFC (AP: +4.2 mm, ML: ±0.75 mm, DV: −3.9 mm) and fixed as previously described. LFP recording and stimulation-synchronous recording were performed using an AlphaLab SnR system. Signals were filtered with a 1 Hz high-pass and 10 kHz low-pass hardware filter, amplified 20-fold, and digitalized at 44 kHz. The stimulation output was connected to the electrode implanted in the brain (negative) and the copper wire (positive). Symmetrical bilateral phasic stimulation waveform was applied with amplitude of 200 μA, pulse width of 60 μs, and frequency of 6 Hz or 130 Hz. Stimulation artifacts were removed via irregular sampling method [2]. Baseline draft and DC component were eliminated with a 2 Hz high-pass filter. High-frequency noises were suppressed by a 100 Hz low-pass filter. LFP power spectral density (PSD) was estimated via

Welch's method with 2 s time window and 1 s overlap. Spectra were normalized by dividing by the integration of PSD from 2 Hz to 90 Hz. Time-frequency analysis was conducted by short-time Fourier transform. Finally, PSDs during 6 Hz and 130 Hz stimulation were separately compared to non-stimulation conditions using cluster-based *t*-tests.

Simultaneous electrical neural stimulation and multimodal MRI

Two symmetrical bundles of MFEs, each consisting of two MFEs (~40-μm diameter), were connected to a customized 4-channel flexible printed circuit (FPC). The FPC was fabricated by a commercial vendor following standard industrial FPC manufacturing processes. Briefly, copper circuits (thickness of 12 μm) were defined on both sides of polyimide-based flexible substrate (thickness of 25 μm) through laser direct imaging photolithography and etching, followed by coverlay lamination (thickness of 12.5 μm) for insulation. Gold plating is applied to solder pad surfaces.

Then the FPC was interfaced with an AlphaLab SnR preamplifier (AlphaOmega, Israel) via a connector (FH34SRJ-8S-0.5SH 50, HIROSE Electric Group) and MRI-compatible cable (Wuhan Greentek Pty. Ltd.). MFEs were bilaterally implanted in rat mPFC (AP: +4.2 mm, ML: ±0.75 mm, DV: −3.9 mm) using the stereotactic method previously described. Ceramic bone anchor screws and dental cement were used to fix the connector and electrode set onto the rat skull. The entire FPC was subcutaneously embedded on the rat's dorsal surface, with only the insertion port exposed.

All MRI experiments were performed on a Bruker 11.7 T scanner (Bruker BioSpec 117/16, Billerica, MA) with a 4-channel surface coil. After connecting the insertion port to the AlphaLab SnR system, the MRI receive coil was positioned over the rat's head. Rats were anesthetized with 1.0–1.5% isoflurane during scanning. Following acquisition of localization and T2W anatomical images, pre-stimulation functional MRI (fMRI), magnetic resonance spectroscopy (MRS) and diffusion-weighted imaging (DWI) were acquired sequentially. Subsequently, 130 Hz stimulation (amplitude of 200 μA, pulse width of 60 μs) was initiated and maintained during fMRI, MRS, and DWI acquisitions. After 15-min washout period, fMRI, MRS, and DWI under 6 Hz stimulation were acquired using identical parameters.

T2W images were acquired using rapid acquisition with refocused echoes sequence with: repetition time (TR)/ echo time (TE) = 3000/6.25 ms, bandwidth = 75 kHz, rare factor = 8, field of view (FOV) = 30 × 30 mm^2, matrix size= 260 × 260, voxel size = 0.115 × 0.115 × 0.5 mm^3, slice thickness = 0.5 mm, number of slice = 45 (no gap). No parallel imaging acceleration was

applied (acceleration factor ≈ 1). The above parameters were also used to evaluate artifacts in T2W acquisitions. Prior to fMRI, 3D shimming was performed. The fMRI scans were performed using a echo-planar imaging sequence with: TR/TE = 2000/13.5 ms, bandwidth = 7102.27 Hz/Px, echo spacing = 0.2816 ms, FOV = 30 × 30 mm^2, voxel size = 0.234 × 0.234 × 0.5 mm^3, matrix = 128 × 128, flip angle = 90°, slice thickness =0.5 mm, number of slice = 50 (no gap), EPI readout direction: left–right (L–R) with phase encoding in the anterior–posterior (A–P) direction, parallel imaging acceleration factor = 2. The above parameters were also used to evaluate artifacts in EPI acquisitions. The DWI was performed with: TR/TE = 2800/17.5 ms, slice thickness = 0.75 mm, number of slice = 32, FOV = 25 × 25 mm^2, matrix size =128 × 128, b-values = 0, 1000 s mm^{-2}. The MRS data were acquired from the mPFC with: TR/TE = 2500/16.2 ms, voxel of interest = 2.5 × 2.5 × 0.75 mm^3.

B_0 inhomogeneity was evaluated by the dual-echo method [3] with: TR = 20 ms, TE_1/TE_2 = 2.139/3.998 ms, FOV = 40 × 40 × 40 mm^3, matrix size = 200 × 200 × 200. B_1 inhomogeneity was evaluated by the double-angle method [4] with: TR/TE = 3000/3 ms, FOV = 27 × 27 × 23 mm^3, matrix size = 90 × 90 × 23, flip angles = 30° and 60°.

MRI data analysis

To quantify electrode artifact size, an automated segmentation pipeline was applied. For each T2W/EPI slice, a rectangular region of interest was manually drawn around the artifact, and the grayscale value of each pixel of the cropped image was normalized to the range [0, 1] using min-max normalization. Then contrast enhancement was performed using a Frangi filter [5] via the fiber metric function from MATLAB's Image Processing Toolbox with a thickness parameter of 9 and "Object Polarity" set to "dark" to highlight hypointense tubular structures. The enhanced image was then binarized using Otsu's method [6], which automatically determines the artifact threshold by maximizing inter-class variance. Artifact size was expressed as the ratio of artifact pixels to total brain pixels within the same slice. Three slices with the largest artifact areas were selected for artifact identification, with the highest pixel ratio designated as the final artifact pixel ratio for comparative analysis.

fMRI data were processed using SPM12 (http://www.fil.ion.ucl.ac.uk/spm/), FSL (FMRIB Software Library), DPABI (A toolbox for Data Processing & Analysis for Brain Imaging) on MATLAB R2023a, ITK-SNAP (http://www.itksnap.org/) and ANTs (Advanced Normalization Tools). After the image format conversion, the mask of rat brain was manually extracted using ITK-SNAP. Skull stripping was performed with FSL. Preprocessing included scaling the voxel size by a factor of 10, motion correction, slicing-timing correction and normalization to the SIGMA rat brain atlas (https://www.nitrc.org/projects/sigma). Band-pass filtering within a

frequency window of 0.01–0.1 Hz was performed with DPABI. Each brain region's ReHo values were then calculated. The statistical analysis was conducted using IBM SPSS Statistics software (version 25.0, IBM, Armonk, NY).

ADC values were calculated using the following equation voxel-by-voxel:

$$\mathrm{ADC} = \frac{\ln(S_1/S_2)}{b_2 - b_1}$$

where S_1 and S_2 denote signal intensities at b=0 and b=1000 s mm^{-2}, respectively. ADC maps were nonlinearly registered to the standardized rat brain atlas (Waxholm Space atlas of the Sprague Dawley rat brain, https://www.nitrc.org/projects/whs-sd-atlas) algorithm implemented in SPM12. Atlas-defined brain regions were propagated to ADC maps for regional value extraction. Two-tailed paired-samples t-tests were performed using IBM SPSS Statistics software. Statistical significance was set at $P < 0.05$.

MRS data were processed with Linear Combination Model by fitting acquired spectra to a basis set of reference spectra. The concentrations of N-acetylaspartate (NAA), creatine (Cr), glutamic acid (Glc), glutamate (Glu), gamma-aminobutyric acid (GABA), aspartic acid (Asp), and taurine (Tau) were analyzed. Between-group comparisons used two-tailed paired-samples t-tests using IBM SPSS Statistics software with a significance threshold set at $P < 0.05$.

1. Li X, Nie Y, Niu Q *et al*. Abnormal prefrontal neural oscillations are associated with social deficits in MECP2 duplication syndrome. *Neurosci bull* 2022; **38**: 1598–602.
2. Nie Y, Guo X, Li X *et al*. Real-time removal of stimulation artifacts in closed-loop deep brain stimulation. *J. Neural Eng.* 2021; **18**: 066031.
3. Schwarzbauer C, Mildner T, Heinke W *et al*. Dual echo EPI – The method of choice for fMRI in the presence of magnetic field inhomogeneities? *NeuroImage* 2010; **49**: 316–26.
4. Cunningham CH, Pauly JM and Nayak KS. Saturated double-angle method for rapid B_1+ mapping. *Magn Reson Med* 2006; **55**: 1326–33.
5. Frangi AF, Niessen WJ, Vincken KL *et al*. Multiscale vessel enhancement filtering. In: Medical Image Computing and Computer-Assisted Intervention—MICCAI'98, Berlin, Heidelberg, 1998; 130–37. Springer Berlin Heidelberg.
6. Otsu N. A threshold selection method from gray-level Histograms. *IEEE Trans Syst Man Cybern* 1979; **9**: 62–6.

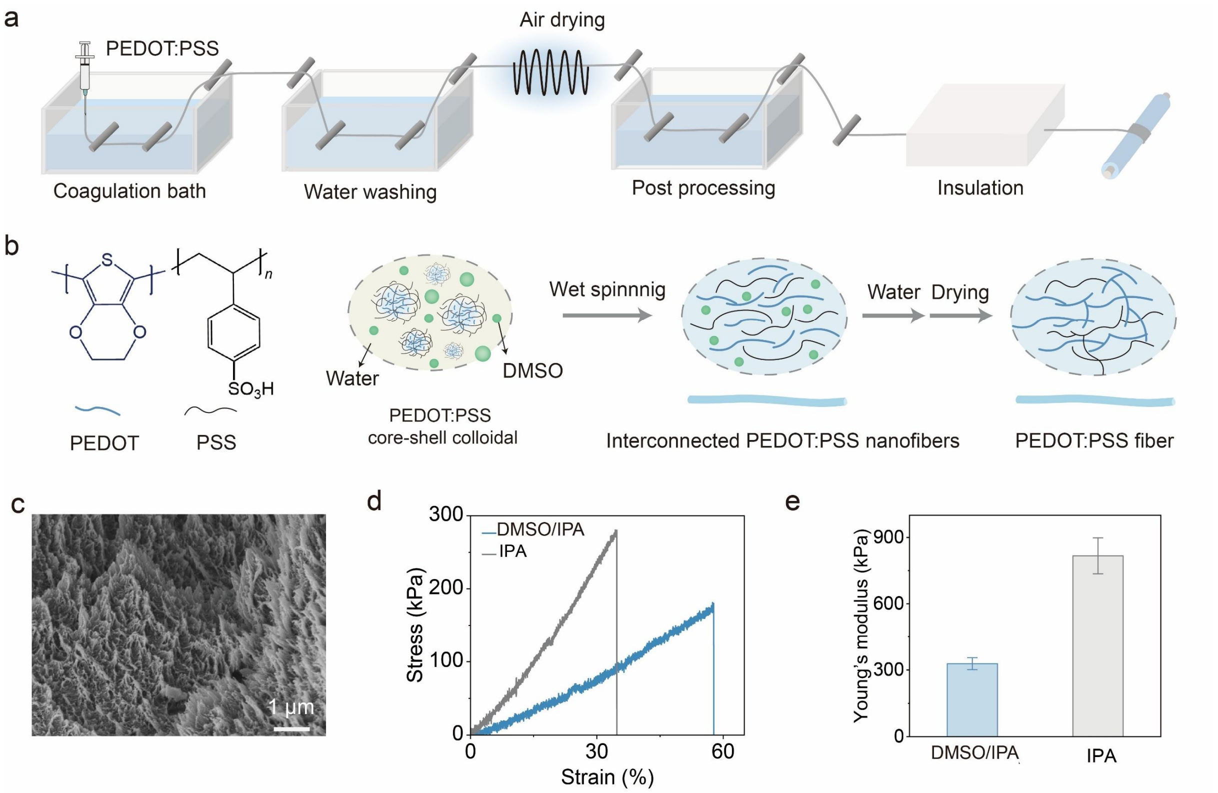

Figure S1. (a) Schematic showing fabrication of PEDOT:PSS fiber including wet spinning, washing, drying, post-processing (liquid infiltration) and insulation. (b) Schematic showing forming process of the PEDOT:PSS fiber. DMSO induces phase separation in PEDOT:PSS microgels, forming extended PEDOT-rich domains. Subsequent coagulation bath immersion drives nanofibrillar bundle assembly into macroscopic fibers. (c) SEM image of the cross-section of lyophilized PEDOT:PSS fiber. (d) Representative stress-strain curve of PEDOT:PSS fiber fabricated in DMSO/IPA co-solvent and pure IPA coagulation baths. Measurements were repeated using five different samples and a typical example was shown. (e) Comparison of Young's modulus for PEDOT:PSS fibers fabricated in DMSO/IPA co-solvent and pure IPA coagulation baths (n = 3, mean ± SD).

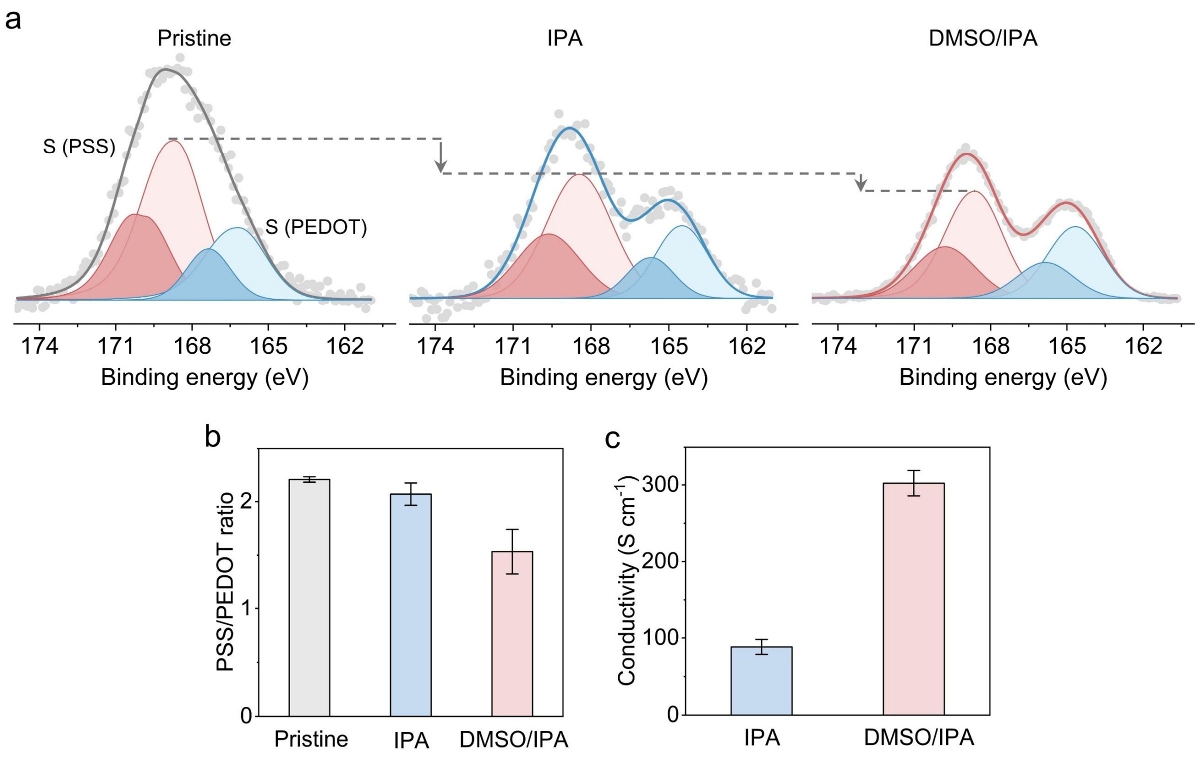

Figure S2. Chemical composition changes in PEDOT:PSS. (a) Sulfur (S) 2p orbital XPS characterizations of pristine PEDOT:PSS suspension, PEDOT:PSS fiber prepared in IPA coagulation bath, and PEDOT:PSS fiber prepared in DMSO/IPA mixed coagulation bath. (b) PSS-to-PEDOT content ratio calculated from XPS peak areas (n = 3, mean ± SD). (c) The conductivities of PEDOT:PSS fibers prepared in IPA and DMSO/IPA mixed coagulation baths (n = 3, mean ± SD).

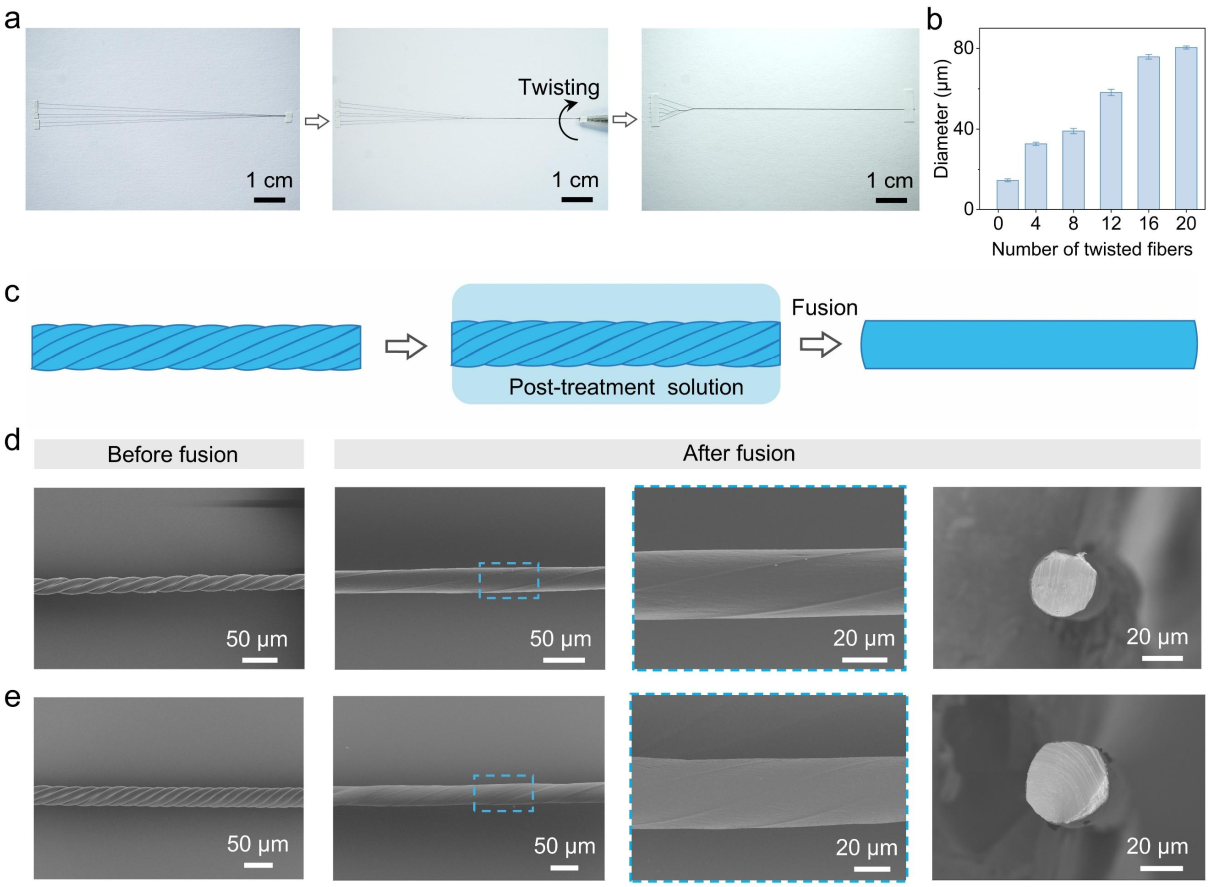

Figure S3. Twist-based assembly of PEDOT:PSS fibers. (a) Photos during the twisting process. (b) Variation in diameter relative to twisting number of PEDOT:PSS fibers (n = 5, mean ± SD). (c) Illustration of the fusion of twist-assembled fibers in post-treatment solution. (d, e) SEM images of 4- (d) and 6-fiber (e) twisted PEDOT:PSS assemblies: side views before/after fusion and cross-sectional views after fusion.

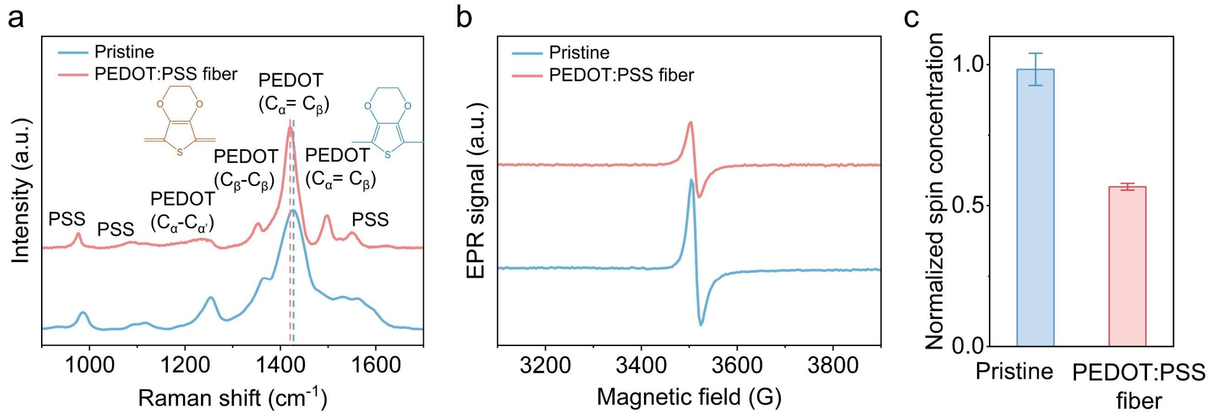

Figure S4. The structure transition of PEDOT:PSS. (a) Raman spectra of pristine PEDOT:PSS and MFE. There was a redshift of the symmetrical $C_\alpha = C_\beta$ stretching vibration of MFE compared to the pristine PEDOT:PSS, illustrating the structure transformation of PEDOT chain from benzenoid to quinoid. (b) Electron paramagnetic resonance (EPR) spectra of pristine PEDOT:PSS and MFE. The reduced EPR intensity of MFE indicates the transition from polarons with a spin of ½ to spinless bipolarons. (c) Quantitative comparison of spin concentration of pristine PEDOT:PSS and MFE (n = 3, mean ± SD).

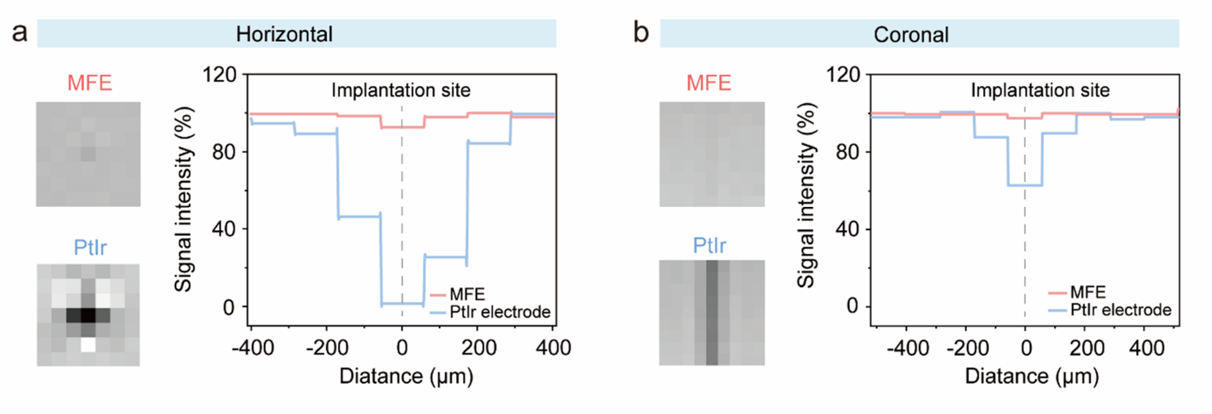

Figure S5. Representative one-dimensional signal intensity profiles (expressed as a percentage of the undisturbed local signal) profiles through the center of the most hypointense voxel. (a) Signal intensity profile of T2-weighted (T2W) image at horizontal section. (b) Signal intensity profile of T2W image at coronal section. Measurements were repeated using three different samples and a typical example was shown.

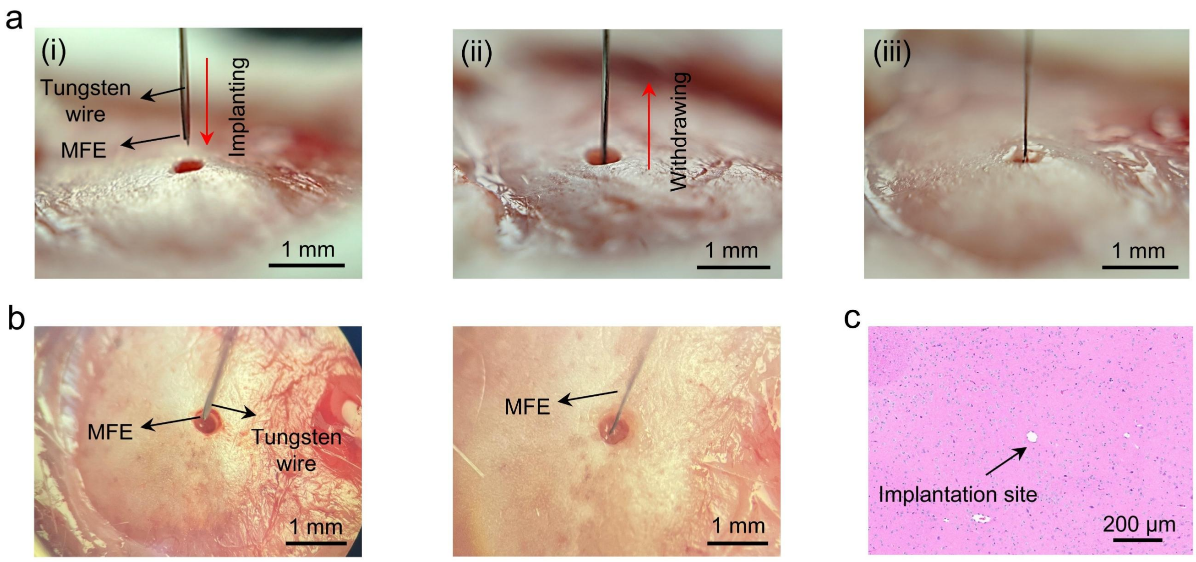

Figure S6. Minimally invasive implantation of MFE. (a) Photographs depicting the tungsten wire-guided MFE implantation procedure. (b) Photographs depicting tissue injury profiles before and after the process of implantation. (c) H&E-stained sections of brain tissue at the electrode implantation site.

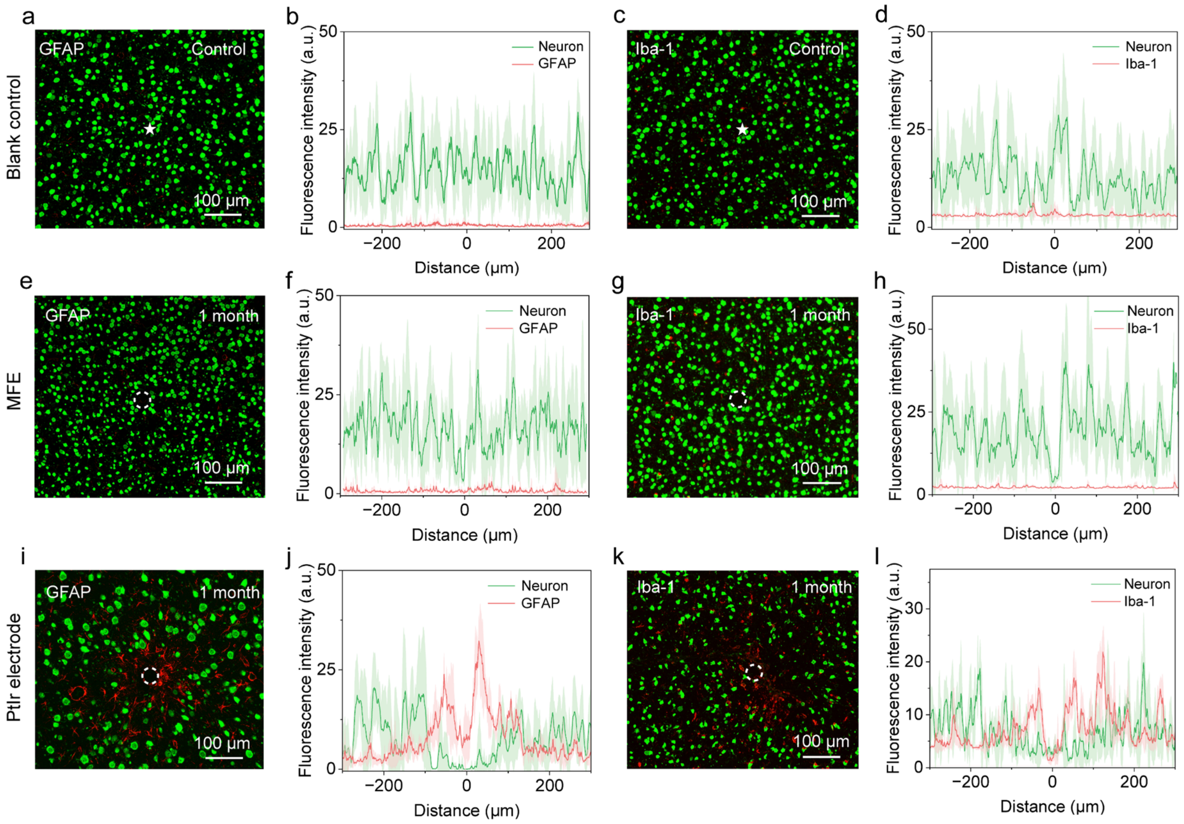

Figure S7. Biocompatibility characterization of the MFE. (a–d) Merged immunohistochemical fluorescence images (a, c) and fluorescence intensities of glial cells and neurons (b, d) surrounding the central point (indicated with a white star) of rat brain slices at horizontal sections in the blank control group. The fluorescence intensity was calculated as mean fluorescence intensity of cells along six equiangular radial axes extending from the central point, measured across a 600-µm-diameter circular region (n = 4, mean ± SD). The curves indicate the mean value. The shadings indicate SD. (e–h) Merged immunohistochemical fluorescence images (e, g) and fluorescence intensities of glial cells and neurons (f, h) surrounding the MFE of rat brain implanted for 1 month. The dashed circles indicate the implantation sites. (i–l) Merged immunohistochemical fluorescence images (i, k) and fluorescence intensity plots (j, l) surrounding the PtIr electrode of rat brain implanted for 1 month at horizontal sections. Dashed circles indicate the PtIr electrode implantation site. The fluorescence intensity was calculated as mean fluorescence intensity of cells along six equiangular radial axes extending from the central point, measured across a 600-µm-diameter circular region (n = 4, mean ± SD). The curves indicate the mean value. The shadings indicate SD. The astrocytes and activated microglia labelled with GFAP (red) and Iba-1 (red), respectively; neurons were labelled with Neuron (green).

315

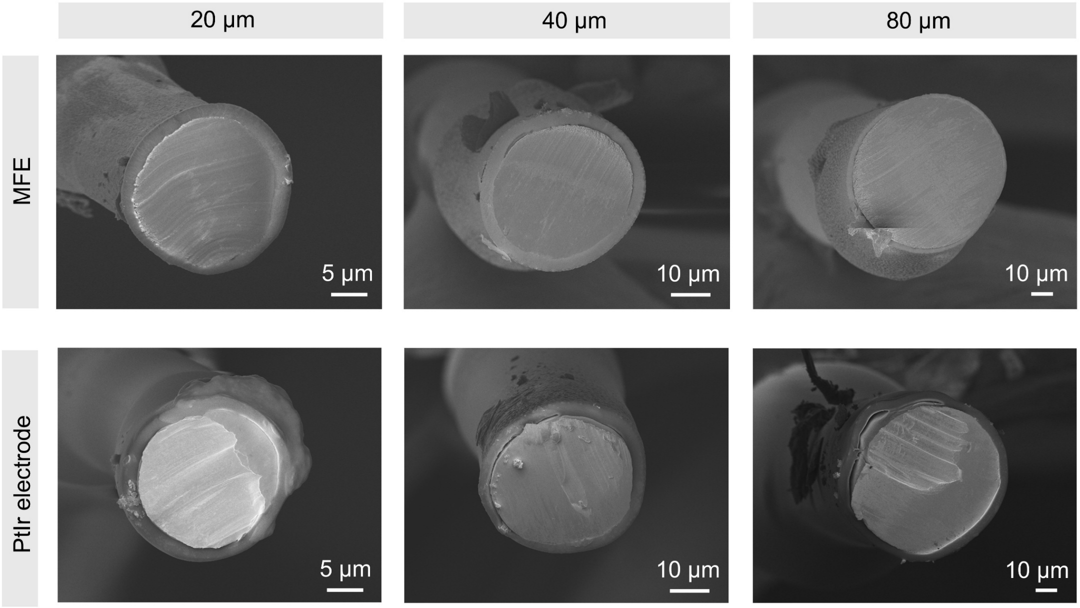

Figure S8. SEM images of MFEs and PtIr electrodes with identical insulation and tip exposure area.

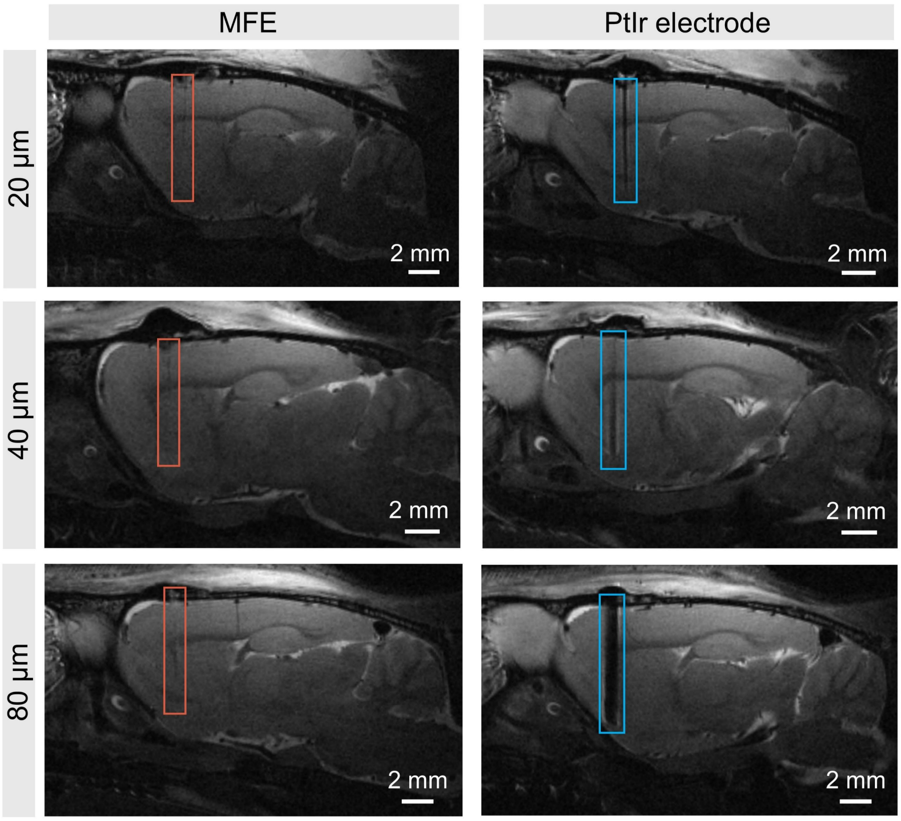

Figure S9. T2W sagittal images of rat brains implanted with electrodes of different diameters and the same implantation orientation.

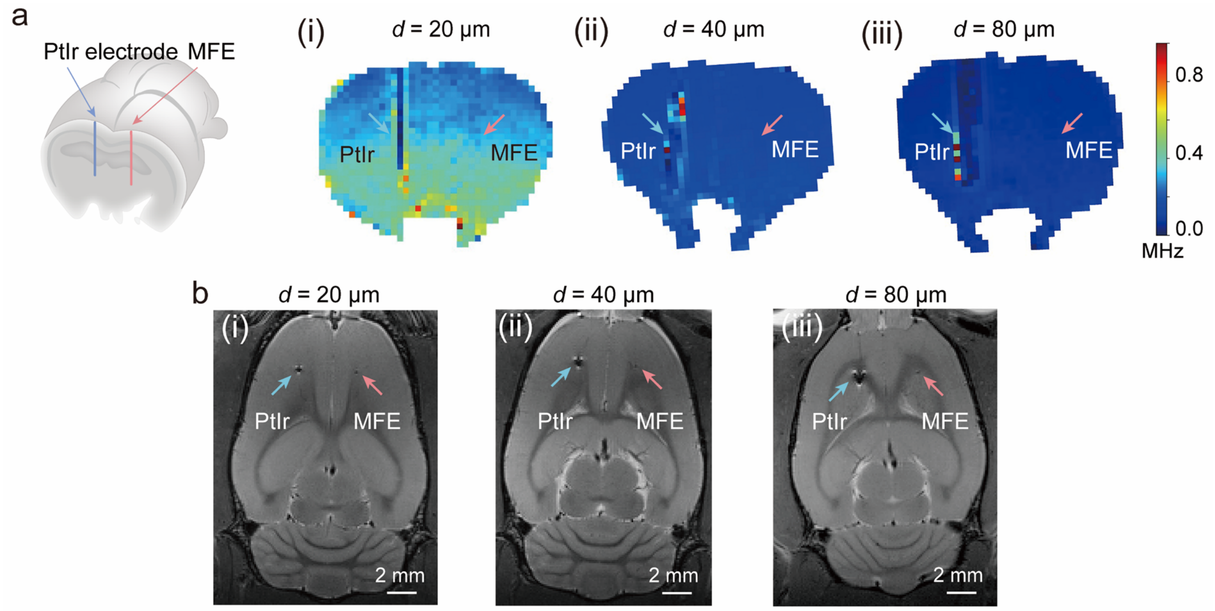

Figure S10. MRI compatibility of the MFE. (a) B_1 distortion maps observed in rats implanted with MFEs and PtIr electrodes with diameters of 20 μm (i), 40 μm (ii), and 80 μm (iii). There were no detectable field distortions around the MFEs with all diameters, whereas PtIr electrodes induced progressively severe B_1 interference with increasing diameter. Blue and red arrows point to PtIr electrodes and MFEs, respectively. Measurements were repeated using four different samples for every diameter and a typical example was shown. (b) Representative T2W images of rat brains implanted with MFEs and PtIr electrodes with diameters of 20 μm (i), 40 μm (ii), and 80 μm (iii), in the horizontal section. Blue and red arrows point to PtIr electrodes and MFEs, respectively.

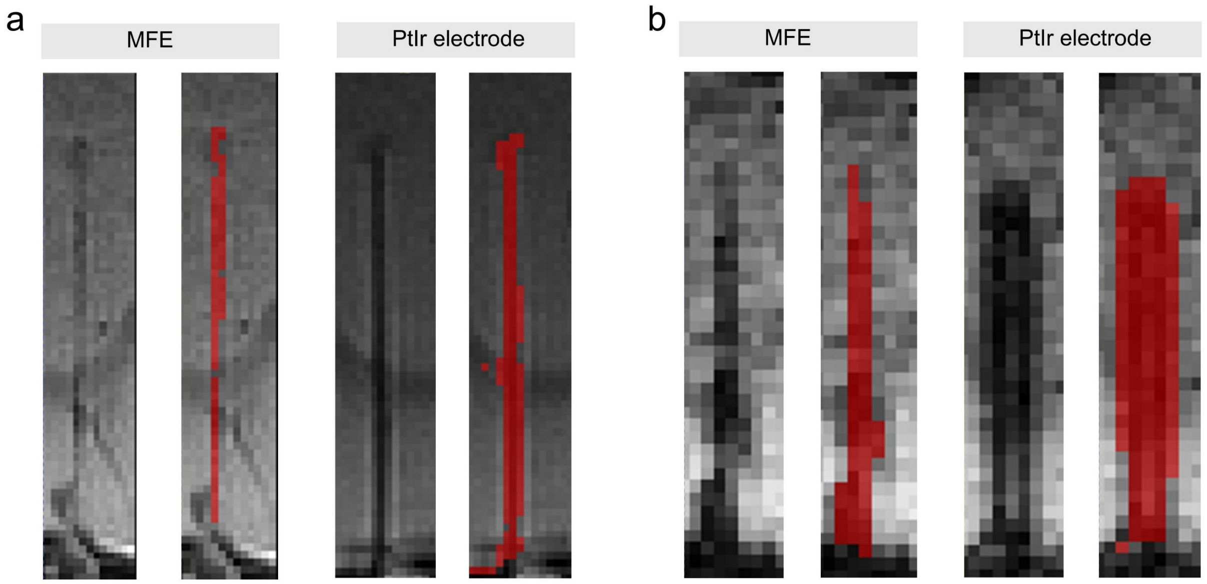

Figure S11. An example showing the automated artifact detection in T2W images (a) and EPI images (b). Right: an original MRI image used for artifact measurement; left: the generated area occupied by electrode artifact. Red areas denote artifact regions.

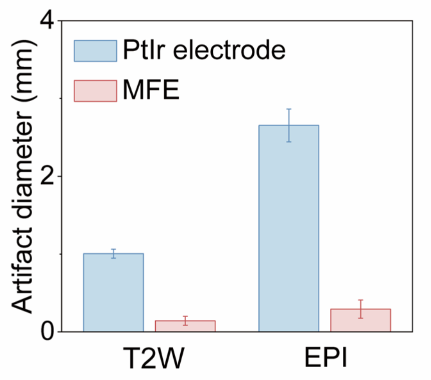

Figure S12. MRI artifact diameter of PtIr electrode and MFE with diameter of 80 μm (n = 4,
345 mean ± SD).

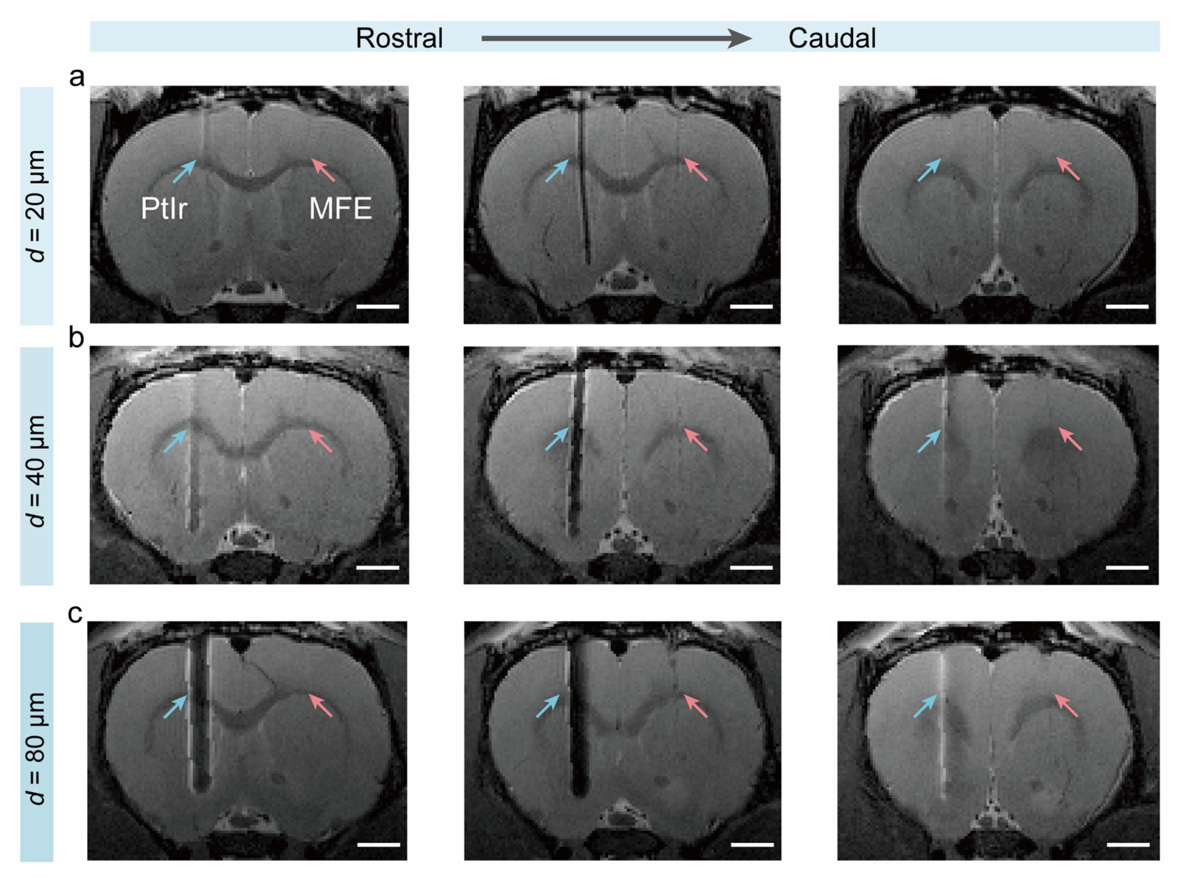

Figure S13. Representative successive T2W MRI slices of electrodes with diameters of 20 μm (a), 40 μm (b) and 80 μm (c) in the rostrocaudal direction. Red and blue arrows point to the MFEs and PtIr electrodes, respectively. Scale bar: 2 mm.

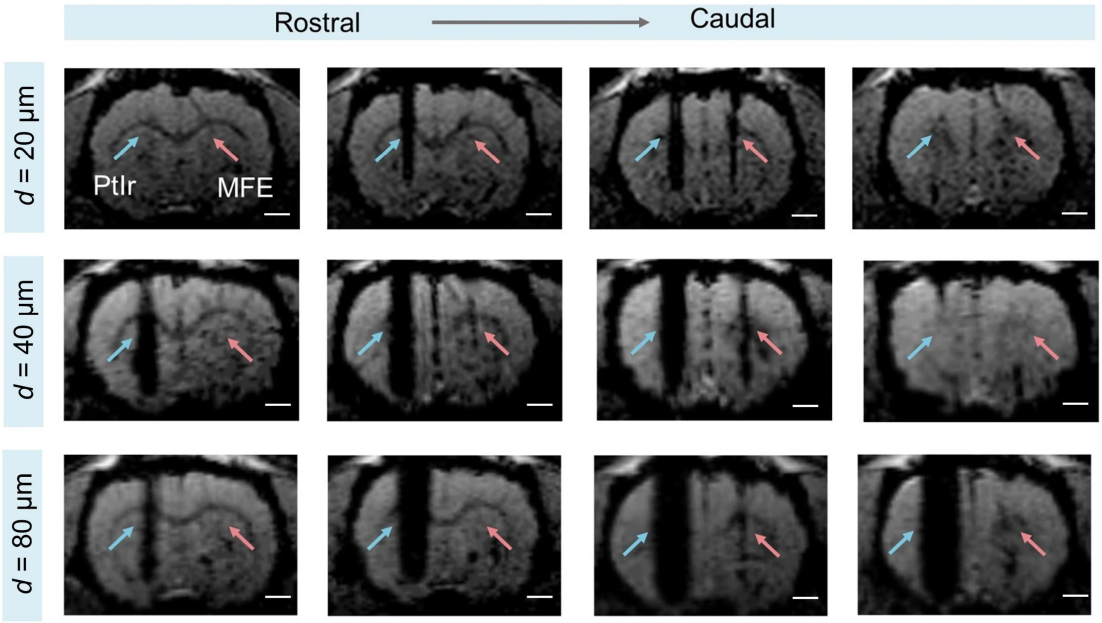

Figure S14. Representative successive echo-planar imaging (EPI) slices of electrodes with diameters of 20 μm (a), 40 μm (b), and 80 μm (c) in the rostrocaudal direction. Red and blue arrows point to the MFEs and PtIr electrodes, respectively. Scale bar: 2 mm.

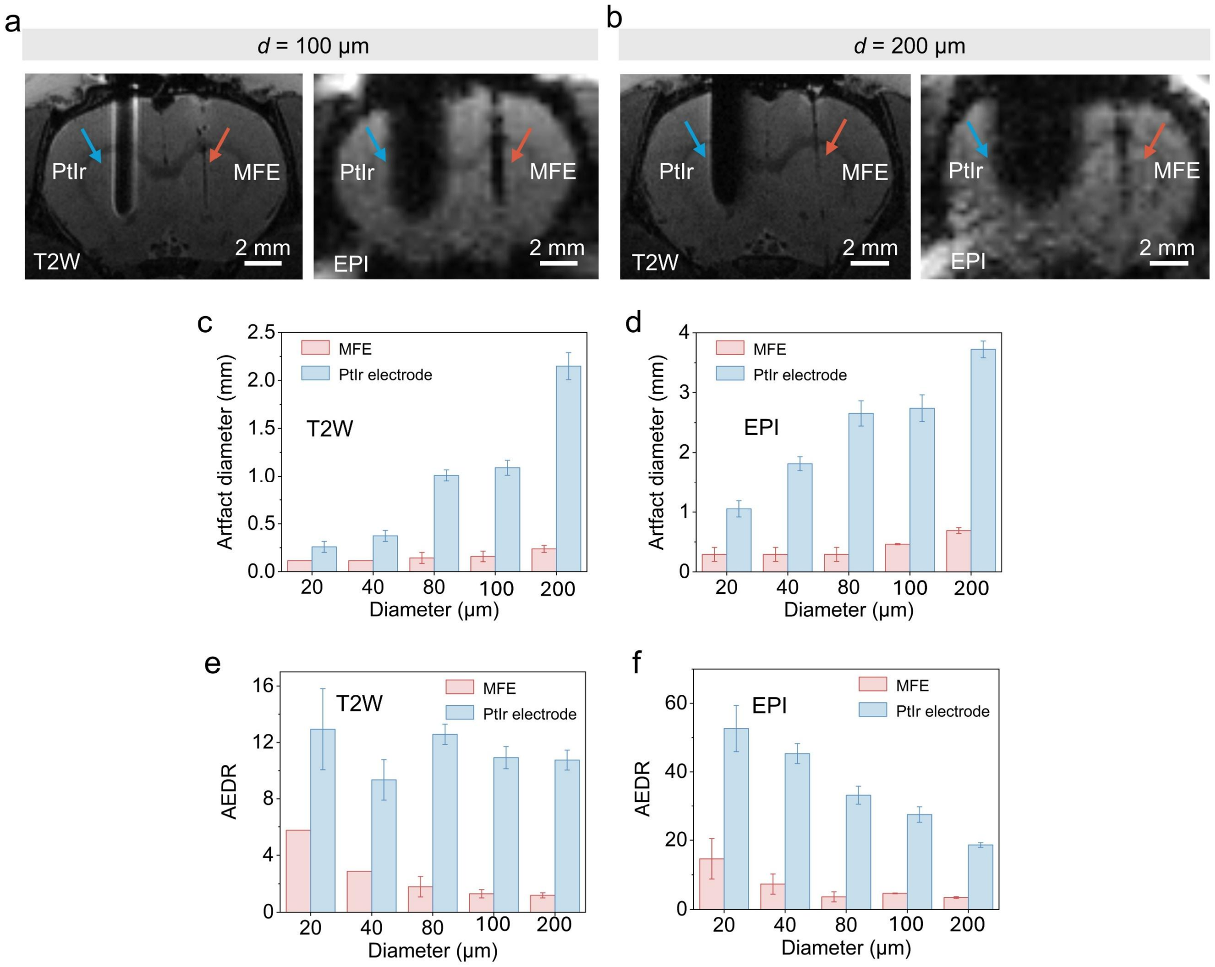

360 **Figure S15.** (a, b) Representative coronal T2W (right) and EPI (left) images of rat brains implanted with MFEs and PtIr electrodes with diameters of 100 μm (a) and 200 μm (b). Blue and Red arrows point to PtIr electrodes and MFEs, respectively. (c, d) The artifact diameter of electrodes with increasing diameters in T2W (c) and EPI (d) images (n = 4, mean ± SD). (e, f) The artifact-to-electrode diameter ratio (AEDR) of electrodes in T2W (e) and EPI (f) images (n
365 = 4, mean ± SD).

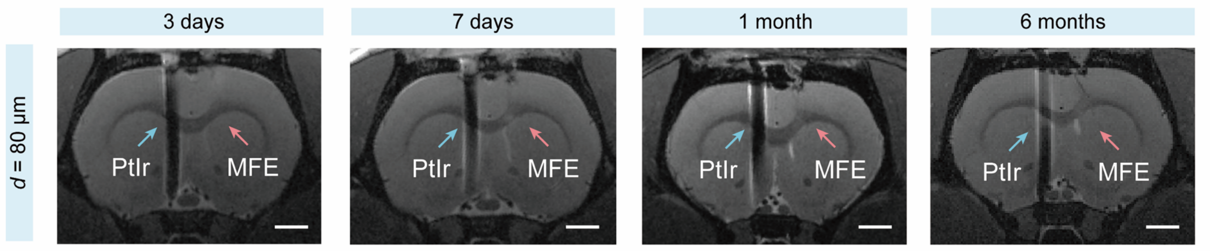

Figure S16. T2W images of rat brain with long-term implanted electrodes. Red and blue arrows point to the MFEs and PtIr electrodes, respectively. Scale bar: 2 mm.

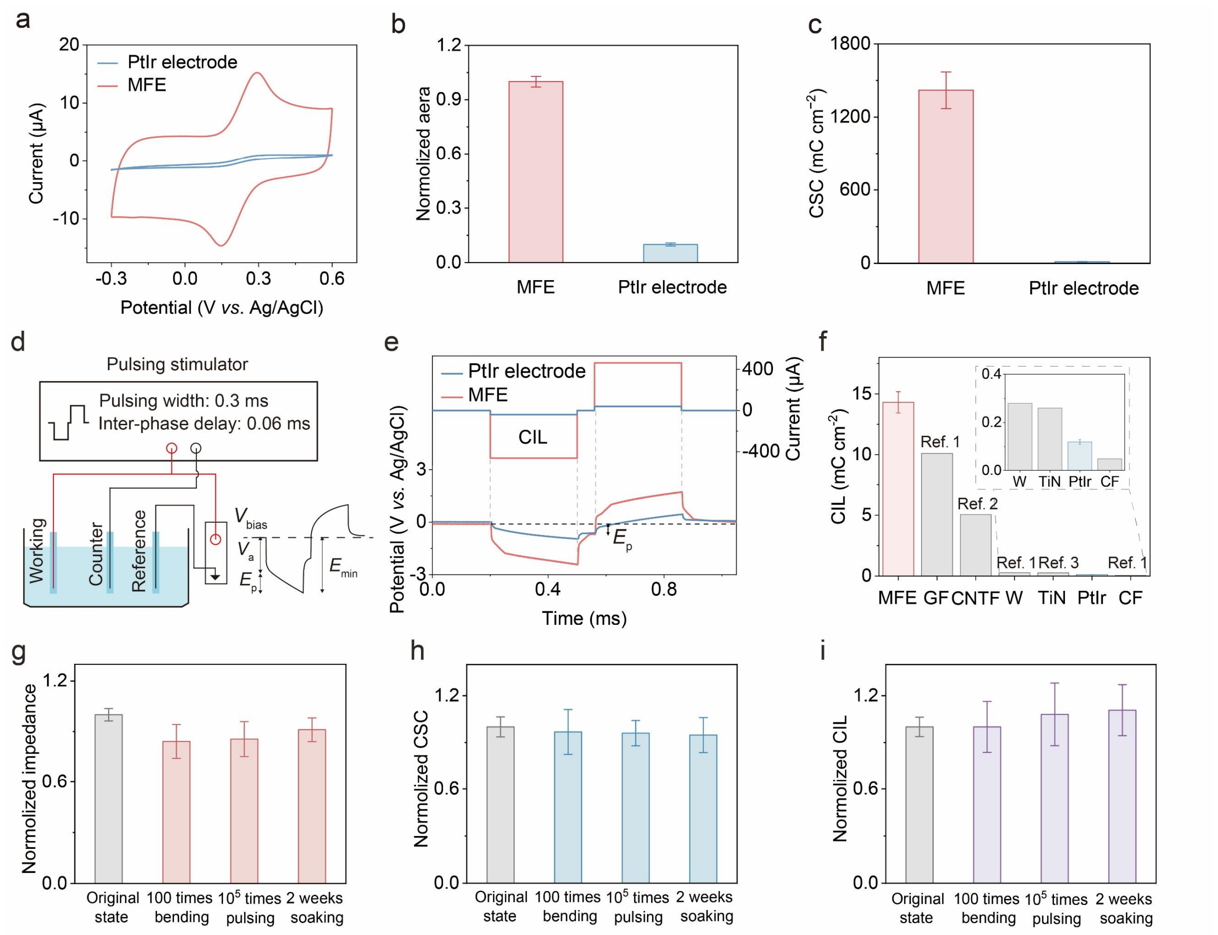

Figure S17. Electrochemical performances and stability of MFE. (a) Cyclic voltammograms of MFE and PtIr electrode in 5 mM potassium ferricyanide solution at a scan rate of 50 mV s^{-1}. (b) Normalized electroactive surface area of MFE and PtIr electrode (n = 5, mean ± SD). (c) Charge storage capacity (CSC) of MFE and PtIr electrode (n = 5, mean ± SD). (d) Schematic diagram of a three-electrode setup for voltage transient measurements. E_p means the interface polarization, which is calculated by subtracting the resistive access voltage (V_a) from the minimum potential (E_{min}). (e) Voltage transients at maximum current pulse driving E_p to water reduction limit (−0.6 V *vs.* Ag/AgCl). (f) Charge injection limit (CIL) of different electrode materials. (n = 5, mean ± SD). GF: graphene fiber; CNTF: carbon nanotube fiber; CF: carbon fiber. (g−i) Impedance at 1 kHz (g), CSC (h), and CIL (i) of MFE after 100 bending cycles, 100,000 current pulses and 2-week phosphate-buffered saline immersion, respectively (n = 5, mean ± SD).

References for Supplementary Figure 16f:

1 Zhao S, Li G, Tong C *et al.* Full activation pattern mapping by simultaneous deep brain stimulation and fMRI with graphene fiber electrodes. *Nat Commun* 2020; **11**: 1788.

2 Lu L, Fu X, Liew Y *et al.* Soft and MRI Compatible Neural Electrodes from Carbon Nanotube Fibers. *Nano Lett* 2019; **19**: 1577−86.

3 Weiland J D, Anderson D J, Humayun M S. In vitro electrical properties for iridium oxide

versus titanium nitride stimulating electrodes. *IEEE Trans Biomed Eng* 2002; **49**: 1574−79.

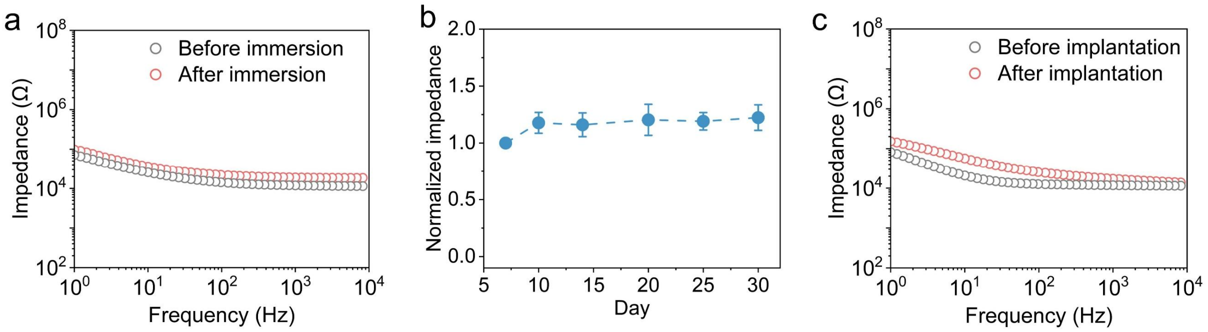

Figure S18. Impedance stability of MFE. (a) Impedance spectra of MFE before and after immersion in a 10 mg mL^{-1} bovine serum albumin solution for 1 h. (b) *In vivo* impedance trajectory of MFE over one month (n = 3, mean ± SD). (c) Impedance spectra of MFE before and after implantation in the brain tissue for 1 month.

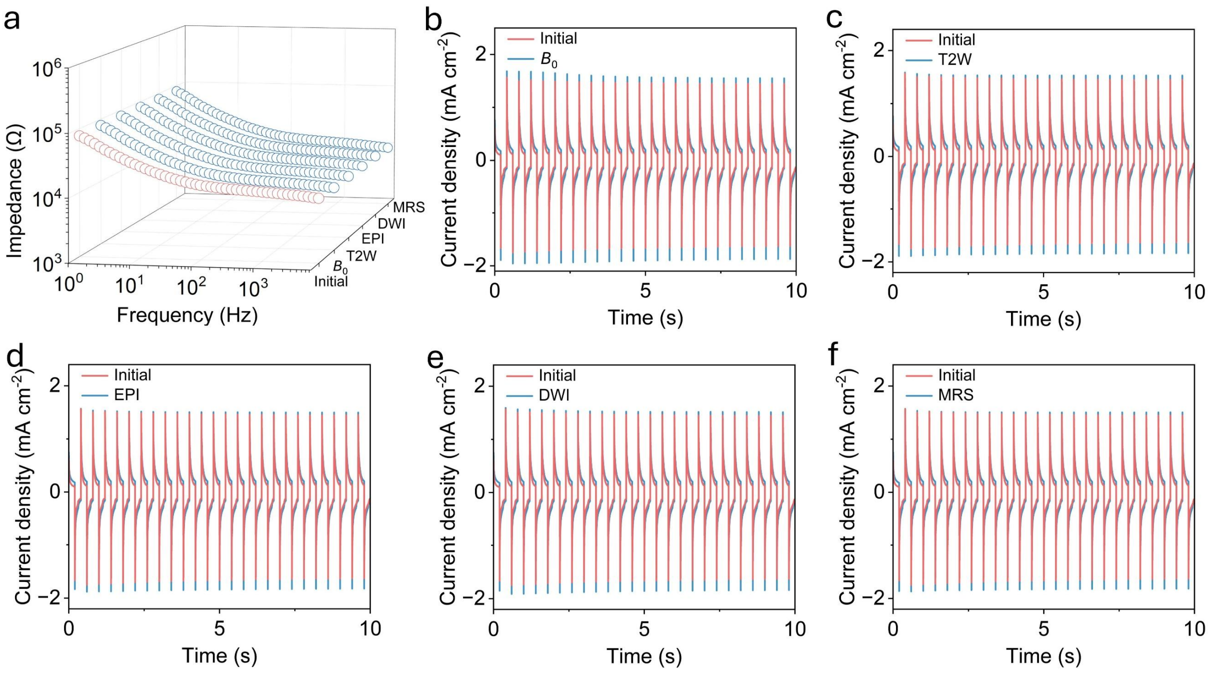

Figure S19. The electrochemical stability inside the MRI bore. (a) The EIS of MFE obtained with no magnetic field (initial) and during MRI scanning with different sequences. (b−f) The current responses of MFE under voltage stimulation of ± 0.5 V with no magnetic field (initial) and different MRI conditions including only static field (b), T2W imaging (c), EPI (d), DWI (e), and MRS (f).

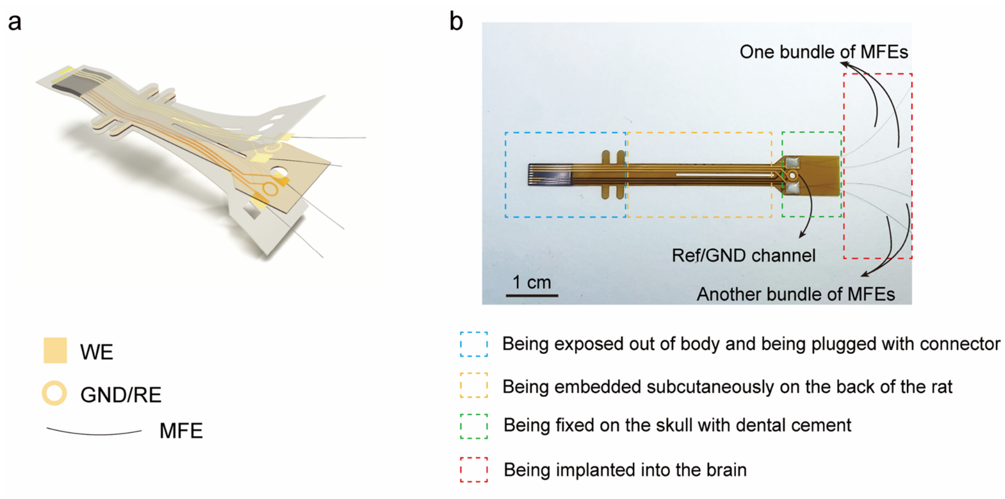

Figure S20. Schematic illustration (a) and photo (b) showing MRI-compatible neural device with MFEs and a customized flexible printed circuit transferring the connector between electrode and external circuit from the cranial region to the dorsal surface. A typical four-channel neural device is shown here. WE: working electrode; GND: ground electrode; RE: reference electrode. The device featured the following layered structure from top to bottom: upper polyimide (PI) coverlay, copper circuit, intermediate PI substrate, copper circuit, and lower PI coverlay. At the implantable end, the centrally positioned annular conductive pad is threaded through the bone screw during implantation to establish electrical contact. Laterally positioned on both front and back sides of the circuit board, four rectangular conductive pads are each connected to a corresponding MFE via silver epoxy adhesive. Dual implantation bundles were designed for bilateral implantation into the medial prefrontal cortex of rats.

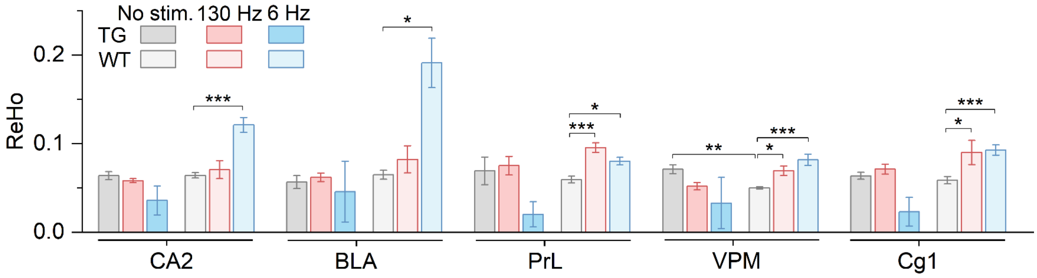

Figure S21. Quantification of ReHo values in specific brain areas of TG and WT rats, with no stimulation, 130 Hz stimulation and 6 Hz stimulation, respectively. (mean ± SEM; $n = 5$ for TG, $n = 6$ for WT; two-tailed t-test; $*P < 0.05$, $**P < 0.01$, $***P < 0.001$). Unlabeled bars indicate no significant difference (ns, $P > 0.05$). CA2: field CA2 of the hippocampus; BLA: anterior part of basolateral amygdaloid nucleus; PrL: prelimbic cortex; VPM: ventral posteromedial thalamic nucleus; Cg1: area 1 of cingulate cortex.

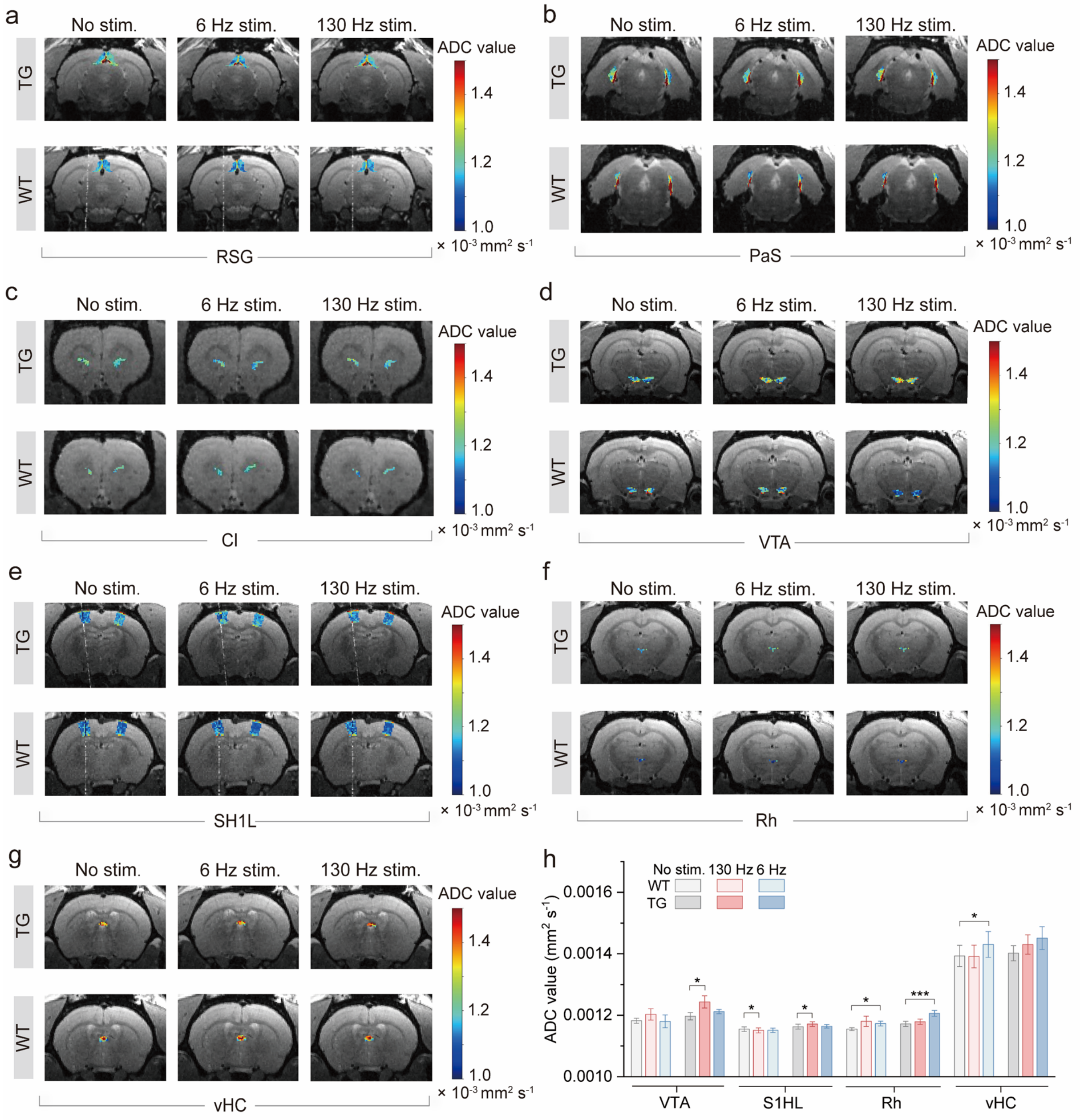

Figure S22. Representative apparent diffusion coefficient (ADC) maps of RSG (a), PaS (b), Cl (c), VTA (d), SH1L (e), Rh (f), vHC (g) regions. Color bar denotes ADC values. (h) ADC values in specific brain areas of TG and WT rats with no stimulation, 130 Hz stimulation, and 6 Hz stimulation, respectively. (n = 7 for TG, n = 8 for WT; mean ± SEM; two-tailed paired sample t-test; *P < 0.05, **P < 0.01, ***P < 0.001). Unlabeled bars indicate no significant difference (ns, P > 0.05). RSG: retrosplenial granular cortex; PaS: parasubiculum; Cl: claustrum; VTA: ventral tegmental area; S1HL: hindlimb region of the primary somatosensory cortex; Rh: rhomboid thalamic nucleus; vHC: ventral hippocampal commissure.

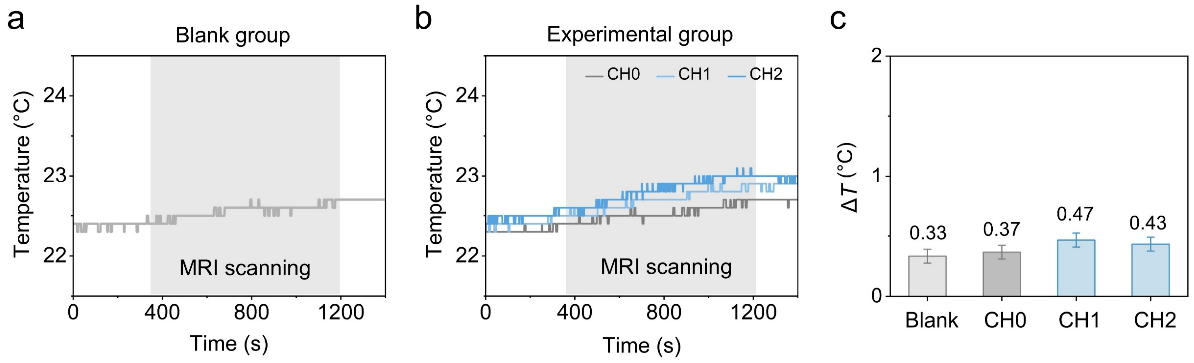

440

Figure S23. *In vitro* radio frequency-induced heating measurements with fiber optics. (a, b) Time-dependent temperature measurements of the blank group with no implants at the test site (a) and experimental group with implanted MFEs (b) during 11.7 T-MRI operations for 15 min with consecutive fMRI, DWI and MRS. The temperature was recorded every 1 second. (c) The

445 change of temperature (ΔT) for different channels (n = 3, mean ± SD). Channel 0 was a built-in temperature reference sample placed away from the implantation site. Channels 1 and 2 were adhered to their respective MFE with ends aligned flush.

Table S1.

Young's modulus and susceptibility difference relative to brain tissue ($|\chi-\chi_{\text{Brain tissue}}|$) for the electrode materials utilized in MFE and previously reported neural electrode materials utilized in representative reported MRI-compatible electrodes and the static magnetic filed (B_0) strength applied for their respective MRI imaging.

**Electrode type**	**Electrode material**	**Young's Modulus (Pa)**	**$\|\chi-\chi_{\text{Brain tissue}}\|$ (ppm)**	**B_0 (T)**	**Neural stimulation/recording-MRI study**	**Ref.**
Deep brain electrode	Brain tissue	100 to 10000	0	/	/	[1]
	Graphene	1.10E+10	0.75	9.4	Yes	[2-4]
	Graphene on polyimide	2.50E+09	/	9.4	No	[5]
	Carbon fiber	2.340E+11	12.75	9.4	Yes	[6-8]
	Carbon nanotube (CNT)	6.00E+8 to 3.00E+10	16.95	7.0	No	[9]
	CNT on SiO_2	1.65 E+11	/	7.0	Yes	[10]
	CNT fiber encapsulated in polycarbonate	2.00E+9 to 2.40E+9	0.95	9.4	Yes	[11]
	Cu (Graphene encapsulated Cu)	1.10E+11	0.55	7.0	No	[12, 13]
	Tungsten	3.90E+11	86.25	9.4	Yes	[12, 14]
	PtIr	1.04E+11	240.05	7.0	Yes	[15]
	Indium/W encapsulated in polycarbonate and cyclic olefin copolymer	2.00E+9 to 2.40E+9	/	9.4	No	[16]
	MXene/PEDOT-PSS	1.22E+10	/	9.4	No	[17]
	**PEDOT:PSS**	**3.28E+5**	**0.18**	**11.7**	**Yes**	**This work**
ECoG electrode	PEDOT:PSS on PDMS	/	/	9.4	No	[18]
	Hyaluronic acid/PEDOT:PSS	2.00E+5	/	9.4	No	[19]
	PEDOT-modified carbon	10000	/	1	No	[20]

fabric based on PVA substrate					
PEDOT:PSS on PDMS	/	/	9.4	No	[21]
Graphene on Parylene-C	2.70E+9 to 3.20E+9	/	3	No	[22]

Abbreviation: ECoG: electrocorticography, PC: polycarbonate, COC: cyclic olefin copolymer, PDMS: polydimethylsiloxane.

References for Supplementary Table 1:

1 Lacour S P, Courtine G, Guck J. Materials and technologies for soft implantable neuroprostheses. *Nat Rev Mater* 2016; **1**: 16063.

2 Wang K, Frewin C L, Esrafilzadeh D *et al*. High-performance graphene-fiber-based neural recording microelectrodes. *Adv Mater* 2019; **31**: 1805867.

3 Zhao S, Li G, Tong C *et al*. Full activation pattern mapping by simultaneous deep brain stimulation and fMRI with graphene fiber electrodes. *Nat Commun* 2020; **11**: 1788.

4 Yu Y, Qiu Y, Li G *et al*. Sleep fMRI with simultaneous electrophysiology at 9.4 T in male mice. *Nat Commun* 2023; **14**: 1651.

5 Tu K, Wang L, Chen H *et al*. Multilayer graphene based MRI compatible MEMS neural probes for both stimulation and recording. *Small* 2025; **21**: e06633.

6 Kozai T D Y, Langhals N B, Patel P R *et al*. Ultrasmall implantable composite microelectrodes with bioactive surfaces for chronic neural interfaces. *Nat Mat* 2012; **11**:1065−73.

7 Dunn J F, Tuor U I, Kmech J *et al*. Functional brain mapping at 9.4T using a new MRI-compatible electrode chronically implanted in rats. *Magn Reson Med* 2009; **61**: 222−8.

8 Fu X, Li G, Niu Y *et al*. Carbon-based fiber materials as implantable depth neural electrodes. *Front Neurosci* 2021; **15**: 771980.

9 Lu L, Fu X, Liew Y *et al*. Soft and MRI compatible neural electrodes from carbon nanotube fibers. *Nano Lett* 2019; **19**: 1577−86.

10 Xia J, Zhang F, Zhang L *et al*. Magnetically compatible brain electrode arrays based on single-walled carbon nanotubes for long-term implantation. *Nanomaterials* 2024; **14**: 240.

11 Driscoll N, Antonini M-J, Cannon T M *et al*. Multifunctional neural probes enable bidirectional electrical, optical, and chemical recording and stimulation in vivo. *Adv Mat* 2025; **37**: 2408154.

12 Zhang Y, Le S, Li H *et al*. MRI magnetic compatible electrical neural interface: From materials to application. *Biosens Bioelectron* 2021; **194**: 113592.

13 Zhao S, Liu X, Xu Z *et al*. Graphene encapsulated copper microwires as highly MRI compatible neural electrodes. *Nano Lett* 2016; **16**: 7731−38.

14 Lai H-Y, Albaugh D L, Kao Y-C J *et al*. Robust deep brain stimulation functional MRI procedures in rats and mice using an MR-compatible tungsten microwire electrode. *Magn Reson Med* 2015; **73**: 1246−51.

15 Van Den Berge N, Vanhove C, Descamps B *et al*. Functional MRI during hippocampal deep brain stimulation in the healthy rat brain. *PLoS One* 2015; **10**: e0133245.

16 Antonini M-J, Sahasrabudhe A, Tabet A *et al*. Customizing MRI-compatible multifunctional neural interfaces through fiber drawing. *Adv Funct Mater* 2021; **31**: 2104857.

17 Gou S, Li P, Yang S *et al* High-performance MXene/PEDOT-PSS microscale fiber electrodes for neural recording and stimulation. *Adv Funct Mater* 2025; **35**: 2424236.

18 Cho Y U, Kim K, Dutta A *et al*. MRI-Compatible, transparent PEDOT:PSS neural implants for the alleviation of neuropathic pain with motor cortex stimulation. *Adv Funct Mater* 2024; **34**: 2310908.

19 Kim S D, Park K, Lee S *et al*. Injectable and tissue-conformable conductive hydrogel for MRI-compatible brain-interfacing electrodes. *Soft Sci* 2023; **3**: 18.

20 Oribe S, Yoshida S, Kusama S *et al*. Hydrogel-based organic subdural electrode with high conformability to brain surface. *Sci Rep* 2019; **9**: 13379.

21 Hong J-H, Lee J Y, Dutta A *et al*. Monolayer, open-mesh, pristine PEDOT:PSS-based conformal brain implants for fully MRI-compatible neural interfaces. *Biosens Bioelectron* 2024; **260**: 116446.

22 Bakhshaee Babaroud N, Palmar M, Velea A I *et al*. Multilayer CVD graphene electrodes using a transfer-free process for the next generation of optically transparent and MRI-compatible neural interfaces. *Microsyst Nanoeng* 2022; **8**: 107.
